# Tcirg1 deficiency delays osteoarthritis progression by impairing lysosome acidification and peripheral accumulation in osteoclasts

**DOI:** 10.3389/fcell.2025.1621648

**Published:** 2025-09-09

**Authors:** Rui Sun, Yang Yang, Qi Ma, Gang Wu, Zhibin Lan, Di Xue, Zhirong Chen, Yajing Su, Zhaopu Tuo, Jiangbo Yan, Long Ma, Xin Zhao, Kuanmin Tian, Xiaoxin He, Ye Ma, Xue Lin, Qunhua Jin

**Affiliations:** ^1^ The Third Ward of Orthopaedic Department, Institute of Osteoarthropathy, Institute of Medical Sciences, General Hospital of Ningxia Medical University, Yinchuan, China; ^2^ Ningxia Key Laboratory of Clinical and Pathogenic Microbiology, General Hospital of Ningxia Medical University, Yinchuan, China

**Keywords:** osteoarthritis, Tcirg1, osteoclast, bone resorption, lysosome

## Abstract

**Introduction:**

Osteoarthritis (OA) is a chronic degenerative joint disease characterized by articular cartilage loss and aberrant subchondral bone remodeling. “T-cell immune regulator 1” (*Tcirg1*), which encodes the a3 subunit of the V-ATPase, has been demonstrated to inhibit the formation of large osteoclasts by reducing intracellular calcium oscillations. Mutations in the *Tcirg1* gene sequence have been associated with osteopetrosis by impairing lysosomal transport in osteoclasts. This study aims to assess the impact of *Tcirg1* on OA progression and to explore its therapeutic potential for the disease treatment.

**Methods:**

Proteomic comparison of weight-bearing region (WBR) *versus* non-weight-bearing region (NWBR) of the subchondral bone was performed in 20 OA patients undergoing total knee arthroplasty. OA was then surgically induced in wild-type and Tcirg1-knockout mice by destabilization of the medial meniscus; disease severity and subchondral bone architecture were evaluated by histology and micro-CT. *In vitro*, primary bone marrow macrophages were differentiated into osteoclasts to assess the role of Tcirg1 in osteoclastogenesis, focusing on cell fusion, bone resorption, and lysosome acidification and distribution.

**Results and discussion:**

Proteomic analysis revealed that TCIRG1 was significantly upregulated in the WBR compared to NWBR of subchondral bone in OA patients, with functional enrichment analysis indicating TCIRG1 correlation with lysosome-related biological processes. In the murine OA model, Tcirg1 expression increased in parallel with osteoclast activity, peaking at 4 weeks post-surgery, which coincided with severe subchondral bone loss. *Tcirg1* deficiency in knockout mice delayed OA progression, as evidenced by reduced cartilage damage, improved subchondral bone mass, and decreased osteoclast activity. *In vitro*, Tcirg1 expression increased during osteoclast differentiation, and its knockdown inhibited osteoclast fusion and bone resorption by impairing lysosome acidification and peripheral accumulation.

**Conclusion:**

Tcirg1 regulates lysosome acidification and peripheral accumulation, thereby influencing osteoclast activity in the subchondral bone. Given that *Tcirg1* knockdown was found to slow down the progression of OA, targeting Tcirg1 may serve as a potential therapeutic strategy for treating OA.

## 1 Introduction

Osteoarthritis (OA) is a chronic degenerative joint disease primarily characterized by articular cartilage degeneration and abnormal subchondral bone remodeling, leading to joint dysfunction, pain, and restricted mobility (Hunter and Bierma-Zeinstra, 2019; Katz et al., 2021; Kloppenburg et al., 2025). Recently, subchondral bone remodeling has been shown to be a crucial process in OA, even before cartilage degeneration occurs (Cinque et al., 2018; Hügle and Geurts, 2017). The key factor driving abnormal subchondral bone remodeling is a dysregulated osteoclast activity (Lin et al., 2019; Zhen et al., 2013). Osteoclasts maintain bone homeostasis under normal conditions by adhering to the bone surface, forming an acidic microenvironment via secretory lysosome exocytosis, and releasing protons and degradative enzymes to resorb the bone matrix (Lacombe et al., 2013; Ng et al., 2019). However, osteoclast activity is significantly enhanced in OA. This overactivation increases subchondral bone resorption and microarchitectural disorganization, compromising mechanical stability via RANKL/RANK/OPG imbalance. It also triggers pro-angiogenic (e.g., VEGF) and inflammatory (e.g., TNF-α, IL-1β) factor secretion: VEGF exacerbates remodeling imbalance to accelerate OA (Hu et al., 2021), while TNF-α/IL-1β induce chondrocyte MMPs expression and apoptosis (Boyle et al., 2003). Additionally, OA synovial cells secrete IL-17 to boost osteoclastogenesis, and osteoclast-derived mediators in turn worsen synovitis, forming a pathological crosstalk (Faust et al., 2020; Störch et al., 2016). Therefore, osteoclast activity is an important target for the treatment of OA.

Recently, lysosomal function in osteoclasts has emerged as a key regulator of bone resorption. Osteoclasts use microtubules to transport secretory lysosomes to the cell periphery, where fusion with the membrane of the ruffled border enables the release of degradative enzymes (Jiang et al., 2024). Ras-related protein Rab-7 (Rab7), a small GTPase, mediates lysosome targeting and fusion with late endosomes. In particular, RUFY4 enhances Rab7-‘lysosome-associated membrane glycoprotein 2’ (Lamp2) interaction to promote lysosomal maturation and cathepsin K (Ctsk) secretion, with its knockdown mitigating pathological bone loss (Kim et al., 2024). Mitochondrial–lysosome contact sites influence effector protein binding to Rab7 by modulating Rab7 activation thereby affecting lysosomal dynamics (Wong et al., 2019). Additionally, ‘Pleckstrin homology domain-containing family M member 1’ (Plekhm1) interacts with Rab7 to regulate vesicular trafficking in osteoclasts, and its mutations (e.g., in Y949–R954/L1011–I1018 regions) disrupt lysosomal transport and cause osteosclerosis via NDE1/NDEL1-related pathways (Das et al., 2024; Van Wesenbeeck et al., 2007). ‘Lissencephaly-1 homolog’ (Lis1) forms complexes with Plekhm1, dynein, and dynactin to stabilize microtubule-dependent transport (Ye et al., 2016). Together, these proteins coordinate lysosomal peripheral enrichment for sustained bone resorption. However, the mechanisms driving lysosomal accumulation at osteoclast ruffled borders remain unclear.

“T-cell immune regulator 1” (Tcirg1) is a key genetic factor in infantile malignant osteosclerosis, with mutations identified in most affected patients. It encodes the a3 subunit of V-ATPase—a proton pump comprising V1 (ATP hydrolysis) and V0 (transmembrane proton transport) domains (Frattini et al., 2000; Qin et al., 2012; Sobacchi et al., 2013). The V0 a subunit has four isoforms (a1-a4), with distinct tissue-specific roles: a1 regulates neuronal synaptic vesicle function (Wang et al., 2014; Zhang et al., 2008), a2 is localized to the Golgi apparatus and maintains its acidic environment (Saw et al., 2011), and a3 is critical for osteoclast-mediated bone resorption, and its knockout inhibits osteoclast formation via downregulation of ‘nuclear factor of activated T cells 1’ (Nfatc1) and IP3R2 (Zhang et al., 2020). Beyond bone, a3 facilitates melanoma invasion and regulates pancreatic β-cell insulin secretion (Nishisho et al., 2011; Sun-Wada et al., 2006). The a3 subunit is expressed in gastric parietal cells, where its mutations impair V-ATPase function, leading to reduced gastric acid secretion, impaired calcium absorption, and subsequent hypocalcemia, which in turn causes autosomal recessive osteopetrosis (Sobacchi et al., 2013). Notably, a3 mediates secretory lysosome accumulation at osteoclast ruffled borders by directly interacting with GDP-bound Rab7, promoting lysosomal-plasma membrane fusion and bone resorption (Matsumoto et al., 2018; Nakanishi-Matsui and Matsumoto, 2022). The a4 participates in renal proton transport (Oka et al., 2001). However, the specific mechanism by which a3-Rab7 regulates microtubule structure and downstream factors affecting lysosome peripheral accumulation remains unclear. As for whether Tcirg1 participates in subchondral bone remodeling by coordinating the accumulation of lysosomes at the ruffled border during the pathogenesis of OA, and whether this process can improve the pathological microenvironment of cartilage, there is still a lack of systematic research to date.

Thus, Tcirg1 may play a critical role in bone metabolism and lysosomal function. This study aimed to explore how Tcirg1 regulates osteoclast function by modulating lysosome acidification and peripheral accumulation (a prerequisite for ruffled border formation), thereby regulating subchondral bone remodeling and slowing OA progression. A comprehensive understanding of this process will help elucidate the pathogenesis of OA and offer a theoretical foundation for novel therapeutic strategies.

## 2 Methods

### 2.1 Human tibial plateau specimens of OA

Human tibial plateau specimens were collected from 20 patients who underwent total knee arthroplasty at the General Hospital of Ningxia Medical University and signed an informed consent form. This proteomic cohort of 20 patients meets the criteria for initial differential protein screening and provides statistical validity. Furthermore, we implemented strict inclusion protocols and mass spectrometry quality control to ensure the robustness of the preliminary screening data. These patients were diagnosed with knee OA according to the diagnostic criteria established by the American College of Rheumatology (Altman et al., 1986), wherein patients with OA caused by metabolic diseases and secondary OA, such as traumatic OA, were excluded. Based on gross assessment of cartilage degeneration and anatomical load distribution, two subchondral bone regions were sampled from each tibial plateau: the “weight-bearing region” (WBR), defined as the severely damaged area in the medial tibial plateau (primary load-bearing zone with marked cartilage degradation and subchondral bone remodeling), and the “non-weight-bearing region” (NWBR), referring to the undamaged area in the lateral tibial plateau (non-load-bearing zone with preserved cartilage integrity and minimal subchondral bone changes). The anatomical locations of WBR and NWBR, along with the typical load distribution pattern and cartilage degeneration status in OA patients, are illustrated in Supplementary Figure S1.

### 2.2 Label-free analysis of tissue proteomes using liquid chromatography–tandem mass spectrometry (LC–MS/MS)

Tissue specimens were collected from 20 patients, with samples from each patient divided into WBR and NWBR groups (20 samples per group) to ensure no cross-contamination. After resection, the tibial plateau specimens were immediately rinsed with ice-cold phosphate-buffered saline (PBS) to remove blood and soft tissue debris. For both WBR and NWBR groups, subchondral bone samples were excised using a sterile surgical blade with the following specifications: each specimen measured approximately 0.5 cm × 0.5 cm × 0.5 cm (length × width × depth), encompassing the subchondral bone plate and underlying trabecular bone while carefully avoiding articular cartilage remnants, with the excision procedure performed under sterile conditions. All specimens were snap-frozen in liquid nitrogen within 10 min of excision and stored at −80 °C until protein extraction to minimize protein degradation (Wilson et al., 2021). After protein extraction, trypsinization was performed, and the peptides were separated using high-performance liquid chromatography (HPLC). Subsequent LC–MS/MS analysis was performed using the Easy-nLC 1200 system (Thermo Fisher Scientific, Shanghai, China) with a Q-Exactive mass spectrometer. The data were processed using Proteome Discoverer software (version 2.4). The screening criteria for differentially expressed proteins were |logFC| > 1 and P < 0.05. Kyoto Encyclopedia of Genes and Genomes (KEGG) pathway enrichment analyses were conducted, and data visualization (heatmaps and volcano plots) was performed using the pheatmap and ggplot2 packages in R (version 4.2.2).

### 2.3 Generation and identification of Tcirg1 knockout mice

*Tcirg1* knockout mice heterozygous mice with a pure C57BL/6J genetic background were newly produced and commercialized for this study by Jiangsu Jicui Pharmachem Biotechnology Co., Ltd. (Strain NO. T003854). Wild-type controls with C57BL/6JGpt background were selected for consistency, ensuring minimal genetic confounding factors. Previous studies have reported that homozygous *Tcirg1* knockout mice (e.g., oc/oc mice from The Jackson Laboratory, with a B6C3Fe hybrid background) are lethal (Johansson et al., 2007), which limits their application in long-term studies requiring sustained survival. In contrast, the heterozygous *Tcirg1* knockout mice used here exhibit a viable phenotype, aligning with ethical guidelines for animal research while maintaining functional relevance: their osteoclast dysfunction phenotypes (e.g., impaired bone resorption) are consistent with previously published *Tcirg1*-deficient models (Li et al., 1999; Voronov et al., 2013).

*Tcirg1* knockout were generated using CRISPR/Cas9 technology. A pair of 20 bp gRNA sequences targeting *Tcirg1* was designed, synthesized, and cloned into expression vectors, followed by sequencing verification. Linearized vectors were transcribed *in vitro* using the MEGA shortscript Kit (Thermo Fisher Scientific, Shanghai, China). gRNA and Cas9 proteins were microinjected into C57BL/6JGpt zygotes, which were then implanted into pseudopregnant C57BL/6JGpt females to produce F0 mice. F0 mice were genotyped using PCR and Sanger sequencing. F0 mutants were bred with wild-type mice to produce F1 offspring, which were then genotyped to confirm germline transmission. Positive F1 heterozygotes were identified as carriers of stable mutations.

### 2.4 Mouse model of OA induced by “destabilization of the medial meniscus” (DMM)

Male C57BL/6J mice, both wild type and Tcirg1^+/−^, of 7–8 weeks of age were used in this study (Jiangsu Jicui Pharmachem Biotechnology Co.). Mice were housed in a controlled environment at 25 °C, 45%–55% humidity, and a 12-h light/dark cycle with food and water available *ad libitum*. After 1 week of acclimatization, experimental OA was surgically induced using DMM. The surgical procedure was performed as follows: mice were anesthetized by intraperitoneal injection of pentobarbital (40 mg/kg), with anesthesia depth assessed via the toe-clamping reflex. Aseptic surgery was conducted at the right knee joint as previously reported (He et al., 2024; Tian et al., 2024), by making a 5-mm longitudinal incision on the medial side, bluntly dissecting subcutaneous tissues to expose the patellar ligament, dislocating the patella laterally to visualize the femoral condyles and medial meniscus, and precisely transecting the medial meniscotibial ligament (MMTL) to destabilize the medial meniscus. Sham-operated controls underwent identical procedures without MMTL transection. After repositioning the patella, the joint capsule, fascia, and skin were sutured layer by layer. At 2, 4, and 8 weeks postoperatively, the mice were euthanized by cervical dislocation, and tissues from the heart, liver, spleen, lungs, kidneys, and knee joints were collecte, with knee joint of sham - operated mice at these time points pooled into one group for subsequent analysis.

### 2.5 Cell isolation and culture

Bone marrow macrophages (BMMs) were isolated from the tibias and femurs of euthanized mice. Muscles were removed, and bones were washed with PBS (Cat. No. PB180327; Procell). The marrow was flushed with α-minimal essential medium (α-MEM) (Cat. No. PM150210; Procell) containing 10% FBS (Cat. No. 164210-50; Procell). The solution was filtered through a 70-μm strainer. The filtrate was centrifuged at 300 × g for 5 min (4 °C). The cell pellet was resuspended in α-MEM with 10% FBS, 1% penicillin, and 1% streptomycin (Cat. No. 5070063; Thermo Fisher Scientific).

Cells were seeded in 6-well plates at a density of 1 × 10^6^ cells/well. The plates were incubated at 37 °C in 5% CO_2_. After 24 h, non-adherent cells were removed, and adherent BMMs were cultured in fresh α-MEM containing 10% FBS, 1% penicillin-streptomycin, 30 ng/mL recombinant macrophage-colony stimulating factor (M-CSF) and 100 ng/mL receptor activator of nuclear factor-κB ligand (RANKL) (both purchased from MCE, Monmouth Junction, NJ, USA) to induce osteoclast differentiation. The medium was replaced every 48 h during the differentiation period. Tartrate-resistant acid phosphatase (Trap) staining was performed on days 1, 3, 5, and 7 to assess osteoclast differentiation (Yuan et al., 2015).

### 2.6 Cell transfection

Small interfering (si)RNAs were purchased from Hanbio (Shanghai, China), and thier sequences are shown in Supplementary Table S1. For the transfection protocol, BMMs were plated in six-well plates at a density of 1 × 10^6^ cells/well and allowed to adhere overnight. The culture medium used was α-MEM containing 10% FBS, 1% penicillin, and 1% streptomycin. The next day, non-adherent cells were removed by washing with PBS, and the adherent cells were used for transfection. For transfection, 5 μL of siRNA (20 μM) and 5 μL of Lipofectamine™ 3000 reagent (Cat. No. L3000015; Thermo Fisher Scientific) were diluted in 250 μL of Opti-MEM (Cat. No. 31985062; Thermo Fisher Scientific) and incubated for 15 min. The transfection mixture was then added to 2 mL of the culture medium in each well, resulting in a final siRNA concentration of 50 nM. For transfection efficiency validation, parallel wells were transfected with CY3-labeled negative control siRNA (si-Cy3) under identical conditions. Transfection efficiency was evaluated 24 h post-transfection using fluorescence microscopy combined with ImageJ software. For each well, 3 random fields were captured, with both CY3 fluorescence images (to visualize CY3-positive cells, red fluorescence) and bright field images (to count total cells) recorded. Transfection efficiency was calculated as the percentage of CY3-positive cells relative to the total number of cells in bright field. After 24–48 h of incubation, the cells were harvested for subsequent analysis.

### 2.7 Western blotting (WB)

For protein extraction, BMMs were lysed with M-PER™Mammalian Protein Extraction Reagent (Cat. No. 78505; Thermo Fisher Scientific) supplemented with protease (Cat. No. 78429; Thermo Fisher Scientific) and phosphatase inhibitors (Cat. No. 78420; Thermo Fisher Scientific) and proteins were quantified using the BCA Protein Assay Kit (Cat. No. KGP903; Kaiji Biotechnology). A total of 20–30 µg of protein from each sample was separated using 10% SDS-PAGE and transferred to a PVDF membrane. The membrane was blocked with 5% nonfat milk for 1 h at room temperature and then incubated overnight at 4 °C with the primary antibody. After washing with Tris - Buffered Saline with 0.1% Tween - 20 (TBST), the membrane was incubated with a horseradish peroxidase-conjugated secondary antibody for 1 h at room temperature. The membrane was washed again and developed using an ECL reagent (Cat. No. RM0021; ABclonal). Signals were detected using a chemiluminescence imaging system (Bio-Rad Laboratories, Inc.) and analyzed using the ImageJ software (version 1.48, NIH). The primary antibodies used in this experiment were against Tcirg1 (1:1,000; Cat. No. 12649-1-AP; Proteintech), Lamp2 (1:1,000; Cat. No. MA1-205; Invitrogen), Rab7 (1:1,000; Cat. No. ab137029; Abcam), Ctsk (1:1,000; Cat. No. sc-48353; Santa Cruz), Matrix metalloproteinase 9 (Mmp9; 1:1,000; Cat. No. 10375-2-AP; Proteintech), Nfatc1 (1:1,000; Cat. No. sc-7294X; Santa Cruz), ‘tartrate-resistant acid phosphatase type 5’ (Acp5; 1:1,000; Cat. No. sc-376875; Santa Cruz), ‘dendritic cell specific transmembrane protein’ (Dc-stamp; 1:1,000; Cat. No. A14630; ABclonal), αv integrin (1:1,000; Cat. No. sc-6617-R; Santa Cruz), β3 integrin (1:1,000; Cat. No. 4702; Cell Signaling Technology), Plekhm1 (1:500; Cat. No. 16202-1-AP; Proteintech), Lis1 (1:1,000; Cat. No. sc-374586; Santa Cruz), and β-actin (1:20,000; Cat. No. 66009-1-Ig; Proteintech). The secondary antibodies included horseradish peroxidase-labeled goat anti-rabbit IgG (1:10,000; Cat. No. ab205718; Abcam) and goat anti-mouse IgG (1:10,000; Cat. No. ab205719; Abcam).

### 2.8 Real-time quantitative PCR (RT-qPCR)

Total RNA was extracted from the cells or tissues using the TRIzol reagent (Cat. No. 9109; Takara) according to the manufacturer’s instructions. For cDNA synthesis, 1 µg of RNA was reverse transcribed using the PrimeScript™ RT reagent kit (Cat. No. RR036Q; Takara) according to the manufacturer’s protocol. Quantitative PCR (qPCR) was performed using the ChamQ SYBR qPCR Master Mix (Vazyme, Q311-02) on an iQ5 Real-Time PCR System (Bio-Rad Laboratories). GAPDH was used as the housekeeping gene, and data were analyzed using the 2^−ΔΔCT^ method. All experiments included three biological replicates, and technical replicates were used to ensure data reliability. The primer sequences are listed in Supplementary Table S2.

### 2.9 Co-immunoprecipitation (Co-IP)

Co-IP was performed using the Pierce™ Classic Magnetic IP/Co-IP Kit (Cat. No. 88805; Thermo Fisher Scientific) according to the manufacturer’s protocol. Briefly, 500 µg of whole-cell lysate (pre-cleared with Protein A/G magnetic beads) was incubated overnight at 4 °C with 2 µg of anti- Tcirg1 antibody (Cat. No. sc-293491; Santa Cruz) or 2 µg of anti-Rab7 antibody (Cat. No. R8779; Sigma-Aldrich). Immune complexes were captured with fresh magnetic beads for 1 h at 4 °C, washed three times with IP lysis buffer, and eluted in 2× Laemmli buffer. Eluates were separated by 10% SDS-PAGE, transferred to PVDF membranes, and immunoblotted as described in Section 2.7. Primary antibodies for immunodetection were anti- Tcirg1 (1:1,000; Cat. No. 12649-1-AP; Proteintech) and anti-Rab7 (1:1,000; Cat. No. ab137029; Abcam). The secondary antibodies included horseradish peroxidase-labeled goat anti-rabbit IgG (Cat. No. ab205718; Abcam) and goat anti-mouse IgG (Cat. No. ab205719; Abcam), which were incubated with the membrane at a 1:5000 dilution for 1 h at room temperature. Subsequently, the signals were visualized using enhanced chemiluminescence.

### 2.10 Enzyme-linked immunosorbent assay (ELISA)

Cell culture supernatants were collected and stored at −80 °C until analysis. The concentration of C-terminal telopeptides of type 1 collagen (CTX-1) in the supernatants was measured using the Elabscience® CTX-1 ELISA kit (Cat. No. E-EL-M3023) according to the manufacturer’s instructions. Briefly, the samples were thawed and processed according to the manufacturer’s protocol, and the absorbance was measured to determine the CTX-1 concentrations. All assays were performed in triplicate for each group.

### 2.11 Micro-computed tomography (micro-CT) analysis

The femur and tibia of the mice were carefully dissected, and the surrounding soft tissues were removed. The bones were fixed in 4% paraformaldehyde at room temperature for 48 h. Micro-CT scanning was conducted using a SkyScan 1276 system (Bruker, Belgium) at a resolution of 9 μm. The region of interest for the analysis was defined as the trabecular bone of the tibia, which typically extends from a defined distance below the growth plate. The key metrics included trabecular mineral density (TMD), bone volume/total volume (BV/TV), trabecular thickness (Tb.Th), and trabecular separation (Tb.Sp), which were quantified using CTAn software (Bruker, Belgium). Three-dimensional reconstructions of 2D slices were generated using CTVox software (Bruker, Belgium). All measurements were performed using a minimum of five biological replicates.

### 2.12 Bone resorption pit assay

The bovine bone slices used for the bone resorption pit assay were prepared as follows: Fresh compact bone from bovine femoral diaphyses was sectioned along the longitudinal axis into 4 mm × 4 mm square slices with a thickness of 300 ± 30 μm using a saw microtome, then ultrasonically cleaned for 10 min (repeated 3 times), air-dried naturally, irradiated with ultraviolet light for 4–5 h, immersed in 75% ethanol, and stored at 4 °C until use. Prior to the experiment, the bone slices were rinsed 3–5 times with PBS or culture medium to remove residual ethanol, preventing interference with cell viability, and individually placed into each well of Corning 96-well plates (Cat. No. 4442). BMMs isolated from murine tibias and femurs were seeded onto the wells containing bone slices at a density of 1 × 10^4^ cells/well, followed by induction of osteoclast differentiation in α-MEM supplemented with 10% FBS, 1% penicillin-streptomycin, 30 ng/mL M-CSF, and 100 ng/mL RANKL in a 37 °C incubator with 5% CO_2_, with the culture medium refreshed every 2 days until mature osteoclasts were formed (usually around 7 days of culture). For scanning electron microscopy (SEM) observation, the bone slices were harvested, adherent cells were removed by ultrasonic cleaning with PBS, then fixed in 2.5% glutaraldehyde, dehydrated with gradient ethanol, dried by critical point drying, and sputter-coated with gold before imaging using a Hitachi S-3400N SEM at 10 kV; quantitative analysis was performed by capturing 5 random fields per well at ×400 magnification, and the percentage of resorbed mineral surface area (% resorbed area) was calculated using NIH ImageJ v1.53c software under a unified threshold to evaluate osteoclastic bone resorption function.

### 2.13 Trap and F-actin staining

BMMs were seeded at 1 × 10^6^ cells/well (6-well plates) in complete α-MEM containing 30 ng/mL M-CSF and 100 ng/mL RANKL for 1, 3, 5, or 7 days to induce osteoclast differentiation. For Trap staining, the cells were fixed with 4% paraformaldehyde for 10 min and stained using a Trap staining kit (Cat. No. CTCC-JD005, PH Biotechnology) according to the manufacturer’s instructions. Trap-positive multinucleated osteoclasts were observed under a light microscope. For F-actin staining, the cells were fixed with 4% paraformaldehyde for 10 min, followed by incubation with rhodamine-conjugated phalloidin (F-actin staining kit, Cat. No. CTCC-JD006, PH Biotechnology) for 30 min. Actin ring formation was visualized using a fluorescence microscope, and images were captured accordingly.

### 2.14 Tissue staining

For histological analysis, the knee joints were immobilized in 4% paraformaldehyde at 4 °C for 24 h, subjected to decalcification in 0.5 M EDTA (pH 7.4), enclosed in paraffin, and sectioned into 4-μm slices. These were stained with safranin-O/fast green (SO/FG) and hematoxylin and eosin (H&E) for histological evaluation and Trap staining for osteoclast activity assessment. Trap-stained sections were acquired from the tibial subchondral bone. Five non-overlapping fields per section were randomly selected using CaseViewer software (3DHISTECH). In each field, Trap-positive (purple) signals were isolated with the “colour deconvolution” plug-in in ImageJ, binarized under a uniform threshold, and expressed as the percentage of Trap-positive area relative to the total bone tissue area (Trap^+^ %), which served as an index of osteoclast activity. Cartilage damage was quantified using two scoring systems based on sample type: for clinical OA patient specimens, the medial and lateral regions of subchondral bone were evaluated using the Mankin scoring system (Mankin and Lippiello, 1970), which is well-suited for regional-specific assessments such as local lesions in the WBR/NWBR subchondral bone; for DMM-induced OA mouse models, the Osteoarthritis Research Society International (OARSI) scoring system (Strassle et al., 2010) was used, as it integrates multi-tissue joint lesions, aligns with the “systemic joint degeneration” characteristic of OA in animal models, and features high standardization, facilitating quantification of the overall effects of interventions. Liver, heart, spleen, and lung tissues were preserved in 4% paraformaldehyde, processed in parallel, and stained with H&E. For immunohistochemistry, the tissue sections were deparaffinized, rehydrated, and subjected to antigen retrieval. Endogenous peroxidase activity was inhibited using 3% H_2_O_2_. Sections were incubated overnight at 4 °C with primary antibody Tcirg1 (1:500; Cat. No. 12649-1-AP; Proteintech), followed by incubation with a secondary antibody (HRP peroxidase-conjugated goat anti-rabbit, 1:10,000; Cat. No. ab205718; Abcam) for 30 min at room temperature. Images of all the stained sections were acquired using an optical microscope (Cat. No. CX43; Olympus Corporation) and examined using ImageJ v1.8.0 software.

### 2.15 Immunofluorescence staining and acid staining

Mouse knee joints and osteoclasts were fixed in 4% paraformaldehyde for 15–20 min at room temperature. Tissue sections were permeabilized with 0.2% Triton X-100 in PBS for 10 min. After blocking with 5% bovine serum albumin (BSA) in PBS for 1 h, the sections were incubated overnight at 4 °C with primary antibodies against: Tcirg1 (1:100; Cat. No. 12649-1-AP; Proteintech), Rab7 (1:200; Cat. No. ab137029; Abcam), Acp5 (1:100; Cat. No. sc-376875; Santa Cruz), Lamp2 (1:100; Cat. No. MA1-205; Invitrogen), and α-tubulin (1:100; Cat. No. sc-8035; Santa Cruz). Following primary antibody incubation, the sections were washed three times with PBS and incubated for 1 h at room temperature with the appropriate Alexa Fluor-conjugated secondary antibodies: Alexa Fluor Plus 594 anti-rabbit (Cat. No. A32754; Thermo Fisher Scientific), Alexa Fluor Plus 594 anti-mouse (Cat. No. A32744; Thermo Fisher Scientific), Alexa Fluor Plus 488 anti-rabbit (Cat. No. A32790; Thermo Fisher Scientific), or Alexa Fluor Plus 488 anti-mouse (Cat. No. A32766; Thermo Fisher Scientific). For the osteoclast acidification assay, when osteoclasts reached the stage of mature multinucleated cells (≥3 nuclei per cell) at 3–4 days after induction, the medium was removed from the dish, and the cells were incubated for 1 h with pre-warmed medium containing LysoSensor Green DND-189 (Cat. No. A66463; Thermo Fisher Scientific) (Carrithers et al., 2007). The culture medium was replaced with fresh medium, and the cell nuclei were counterstained with DAPI (Cat. No. R37605 and R37606; Thermo Fisher Scientific). Fluorescence images were acquired using a STELLARIS 5 confocal microscope (Leica), and an image analysis was performed using ImageJ v1.8.0 software.

### 2.16 Statistical analysis

All statistical analyses were performed using GraphPad Prism version 9.0 (GraphPad Software Inc.). Data are expressed as the mean ± standard error of the mean (SEM). For comparisons between two groups, an unpaired two-tailed Student’s *t*-test was used. For comparisons involving three or more groups, ANOVA with Tukey’s *post hoc* test was used to assess statistical significance. Statistical significance was set at P < 0.05. All experiments were independently repeated at least three times.

## 3 Results

### 3.1 Proteomic analysis and validation of clinical samples from patients with OA

**FIGURE 1:**
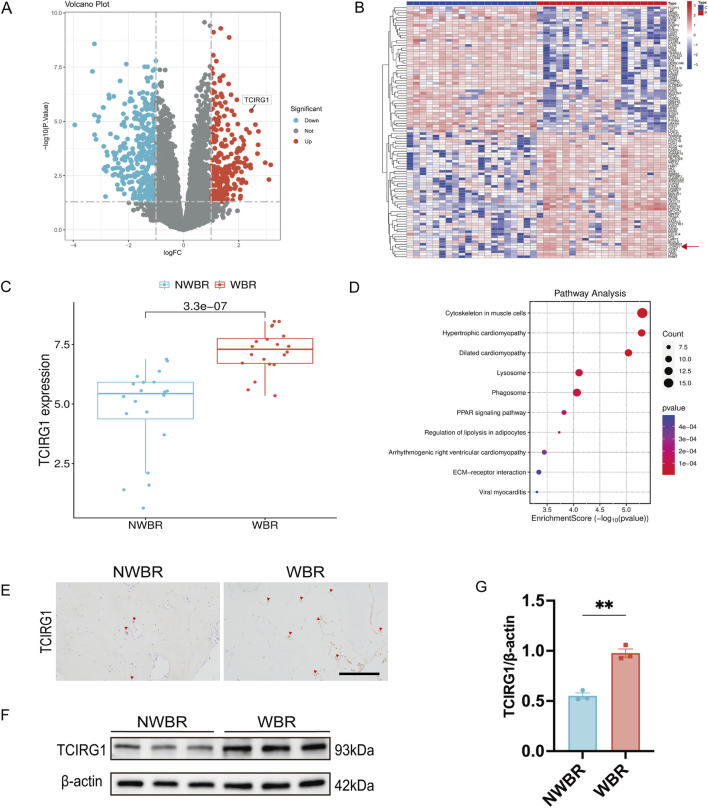
TCIRG1 upregulation in WBR of subchondral bone compared with NWBR in OA patients: proteomic and histological validation **(A)** Volcano plot and **(B)** Heat map of differentially expressed proteins. The red arrow points to TCIRG1. **(C)** Box line plot of TCIRG1 expression. **(D)** Results of KEGG functional enrichment analysis of differentially expressed proteins. The x-axis represents the enrichment score (Enrichment Score), while the color gradient indicates the p-value. Blue denotes higher p-values, whereas red signifies lower p-values. **(E)** Immunoistochemical staining of TCIRG1 in the subchondral bone of representative clinical specimens from a patient with OA. The red triangles represent positive cells. scale bar, 200 μm. **(F)** Western blotting showing the expression of TCIRG1 in the NWBR and WBR of the subchondral bone. **(G)** Quantitative analysis based on **(F)**, data are expressed as mean ± SEM (n = 3 per group). Statistical significance was determined using an unpaired two-tailed Student’s t-test for comparisons between two groups. **P < 0.01.

We excised subchondral bone tissue from the tibial plateau area of 20 patients who underwent total knee arthroplasty to perform proteomics analysis. The weight-bearing region of each patient was designated as the WBR group, and the non-weight-bearing region was designated as the NWBR group (Supplementary Figure S1). The volcano plot revealed that TCIRG1 was significantly upregulated as a differentially expressed protein in WBR compared to the NWBR group (p < 0.05, logFC > 1) (Figure 1A). The heatmap displays the top 100 most significantly altered proteins, which included TCIRG1 (Figure 1B). Box plot analysis confirmed a significant elevation of about 1.3-fold in TCIRG1 expression in the WBR group relative to NWBR (Figure 1C). Subsequent functional enrichment analysis demonstrated significant correlation of the differentially expressed proteins, including TCIRG1, in lysosome-related biological processes based on KEGG pathway analysis (Figure 1D). To confirm the proteomic results, we randomly selected three of the 20 patients’ tibial plateau samples for subsequent analysis. The status of the cartilage and subchondral bone sites in the tibial plateau was determined using safranin O/fast green staining and H&E staining, and Mankin’s score was assigned to assess the severity of OA. The results showed significant cartilage degeneration and subchondral bone remodeling with elevated Mankin’s scores in the WBR group (Supplementary Figure S2). Immunohistochemistry (IHC) showed that TCIRG1-positive cells were present in the subchondral bone, with more positive cells observed in the WBR group than in the NWBR group (Figure 1E), while WB revealed that the protein expression of TCIRG1 in the subchondral bone of the WBR group was 40% higher than that in the NWBR group (Figures 1F,G). These results indicate that TCIRG1 is significantly upregulated in subchondral bone from the WBR compared to the NWBR in patients with OA, with enrichment in lysosome-related biological processes. The concurrent changes in TCIRG1 expression, cartilage degeneration, and subchondral bone remodeling suggest a potential association between TCIRG1 and OA-related pathological processes.

### 3.2 Tcirg1 increased expression is associated with increased osteoclast activity in the subchondral bone of OA mice

**FIGURE 2:**
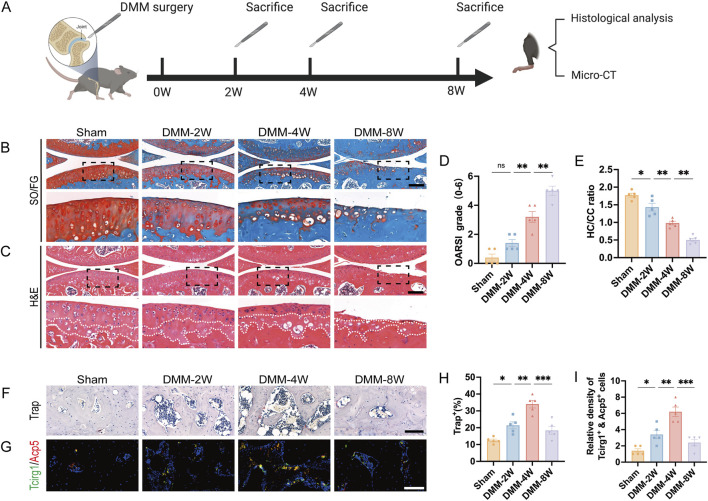
Increased Tcirg1 expression is associated with increased osteoclast activity in the subchondral bone of OA mice. **(A)** Schematic diagram illustrating the animal experiment design. Representative images of **(B)** SO/FG staining and **(C)** H&E staining of the knee joints from the sham and DMM groups at 2, 4, and 8 weeks (W) post-surgery; scale bar: 200 μm. **(D)** OARSI grading. **(E)** Statistical analysis of the hyaline cartilage/calcified cartilage (HC/CC) thickness ratio based on **(C)**. **(F)** Trap staining of the subchondral bone in each group. Scale bar, 100 μm. **(G)** Immunofluorescence staining of Tcirg1 and Acp5 in the subchondral bone from each group. Scale bar, 50 μm. Quantitative analyses of **(H)** percentage of Trap-positive cells, and **(I)** the number of Tcirg1 and Acp5 double positive cells in the subchondral bone. Data are expressed as the mean ±SEM (n = 5). Statistical significance was determined using one-way ANOVA followed by Tukey’s *post hoc* test. *P < 0.05, **P < 0.01, ***P < 0.001; ns, not significant. SO/FG, safranin-O/fast green; H&E, hematoxylin and eosin.

To investigate the relationship between Tcirg1 and osteoclasts in OA, we took advantage of a mouse model of post-traumatic OA to examine the relationship among Tcirg1 expression, OA progression, and osteoclast activity. The model was established via surgical DMM (Figure 2A). The knee joints of the mice were analyzed using SO/FG and H&E staining (Figures 2B,C). In the DMM group, the OARSI score gradually increased with postoperative time (Figure 2D), while the ratio of hyaline to calcified cartilage layers progressively decreased (Figure 2E). These results indicate that the pathological severity of osteoarthritis tends to increase overtime after DMM surgery.

We further analyzed the changes in the number of osteoclasts, representing the bone resorption capacity, in the subchondral bone. Trap staining (Figure 2F) and immunofluorescence analysis (Figure 2G) showed that: the mumber of Trap-positive cells increased significantly at 2 and 4 weeks after DMM, peaking at 4 weeks (1.5-fold increase compared to the sham group, Figure 2H); meanwhile, Tcirg1, which colocalized with the osteoclast marker Acp5 in subchondral bone, also had its positive osteoclast count peak at 4 weeks after DMM (3-fold increase compared to the sham group, Figure 2I). We analyzed subchondral bone changes in each group of mice using micro-CT (Supplementary Figures S3A–C). BV/TV, TMD, and Tb.Th decreased in the 2-week post-DMM group compared to the sham group, whereas Tb.Sp increased. Four weeks after DMM surgery, there was further bone loss in the subchondral bone, with a significant decrease in BV/TV, Tb.Th, and BMD and a significant increase in Tb.Sp compared to 2 weeks after surgery. In contrast, at 8 weeks after DMM surgery, a significant increase in subchondral bone mass was observed compared to the sham group, as evidenced by elevated TMD, BV/TV, and Tb.Th and decreased Tb.Sp. Overall, 4 weeks after DMM surgery was the most severe period of subchondral bone loss (compared to the sham group, BV/TV decreased by 22%, TMD by 8%, Tb.Th by 9%, while Tb.Sp increased by 8%, Supplementary Figures S3D–G), which is consistent with the increase of Trap-positive osteoclasts peaked at 4 weeks after DMM. These results indicate a strong correlation between increased Tcirg1 expression, increased osteoclast activity, subchondral bone resorption, and subsequent cartilage degeneration during the progression of DMM-induced OA.

### 3.3 Tcirg1 deficiency slows the progression of OA in mice

**FIGURE 3:**
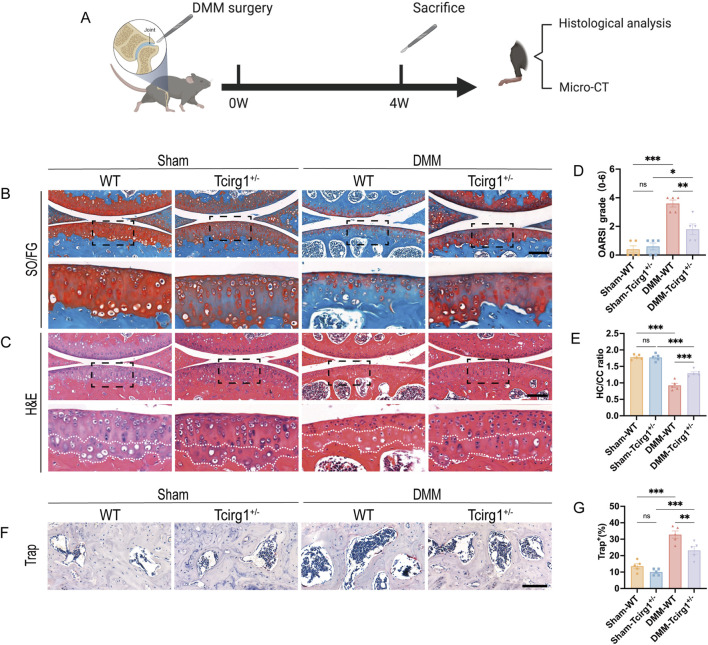
Tcirg1 deficiency slows OA progression in mice. **(A)** Schematic overview of the experimental design with WT and Tcirg1^+/−^ mice. DMM surgery-induced OA was performed in both groups. Representative images of **(B)** SO/FG and **(C)** H&E staining at 4 weeks post-DMM surgery; scale bar: 200 μm. **(D)** OARSI grading for all groups. **(E)** Quantitative analysis of the hyaline cartilage/calcified cartilage (HC/CC) thickness ratio. **(F)** Trap staining in the subchondral bone of the tibia at 4 weeks post-DMM surgery. Scale bar, 100 μm. **(G)** Quantitative analysis of the percentage of Trap-positive cells, data are expressed as the mean ± SEM (n = 5 per group). Statistical significance was determined using one-way ANOVA followed by Tukey’s *post hoc* test. *P < 0.05, **P < 0.01, ***P < 0.001; ns, not significant. SO/FG, safranin-O/fast green; H&E, hematoxylin and eosin.

To investigate the role of Tcirg1 in the pathogenesis of osteoarthritis, we utilized the aforementioned *Tcirg1* ^*+/−*^ mice model, which retains the advantage of long-term survival, thereby allowing us to directly evaluate its impact on OA progression. DMM surgery was performed to induce OA in wild-type and *Tcirg1* ^*+/−*^ mice, while a sham group underwent identical surgical procedures without MMTL transection (Figure 3A). The severity of OA was evaluated using safranin O/fast green staining, H&E staining, and the OARSI scoring system (Figures 3B,C). The results revealed that wild-type mice that underwent DMM surgery exhibited more severe OA in the knee joints than the mice that did not undergo MMTL transection, as evidenced by a significant increase in OARSI score (Figure 3D) and a decrease in the hyaline-to-calcified cartilage layer ratio (Figure 3E). However, these changes were significantly reduced in *Tcirg1* ^*+/−*^ mice, suggesting that *Tcirg1* deletion effectively delays the pathological degeneration in OA.

To further investigate the impact of Tcirg1 on OA subchondral bone, Trap staining was used to analyze the changes in osteoclast numbers in the subchondral bone of *Tcirg1* ^*+/−*^ mice. The results revealed a significant reduction in the number of osteoclasts in the subchondral bone of *Tcirg1* ^*+/−*^ mice 4 weeks after DMM surgery (30% reduction compared to the DMM-WT group) (Figures 3F,G). Subchondral bone changes in each group of mice were further analyzed using micro-CT (Supplementary Figures S4A–C). The results indicated that at 4 weeks after DMM surgery, *Tcirg1* ^*+/−*^ mice exhibited significant increases in BV/TV (20%), TMD (7%), and Tb.Th (8%) and a significant decrease in Tb.Sp (6%) compared to the DMM-WT group (Supplementary Figures S4D–G). Overall, *Tcirg1* knockdown alleviated OA-induced cartilage damage and abnormal subchondral bone remodeling.

To comprehensively assess the potential effects of *Tcirg1* knockout on other organs, we performed H&E staining of tissue samples from the heart, liver, spleen, lungs, and kidneys of adult wild-type and *Tcirg1* ^*+/−*^ mice. The results showed no significant histological differences between the two groups (Supplementary Figure S5). This indicates that *Tcirg1* knockdown did not significantly interfere with the developmental processes of the heart, liver, spleen, lungs, or kidneys in mice.

### 3.4 Tcirg1 exhibits a lysosomal distribution and becomes progressively enriched in the cell periphery during osteoclast differentiation

**FIGURE 4:**
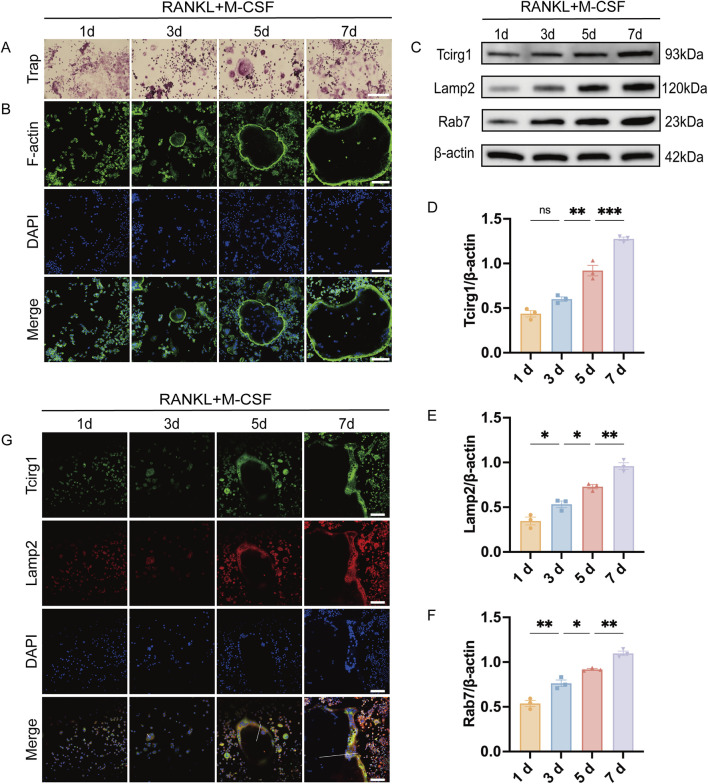
Tcirg1 exhibits lysosomal distribution and progressive enrichment at the cell periphery during osteoclast differentiation. RANKL and M-CSF induced BMMs to undergo osteoclast differentiation that was analyzed at various time points. **(A)** Trap staining and **(B)** immunofluorescence images of F-actin and DAPI. **(C)** WB analysis of Tcirg1, Lamp2, and Rab7 protein expression. Quantitative analysis of **(D)** Tcirg1 protein expression, **(E)** Lamp2 protein expression, and **(F)** Rab7 protein expression. **(G)** Immunofluorescence images of Tcirg1, Lamp2, and DAPI. The white lines extend along the short axis of the cells, from the center to the periphery, to collect and analyze the fluorescence intensity distribution along this path of Tcirg1 and Lamp2 scale bar, 100 μm. Data are expressed as the mean ±SEM (n = 3 per group). Statistical significance was determined using one-way ANOVA followed by Tukey’s *post hoc* test. *P < 0.05, **P < 0.01, ***P < 0.001; ns, not significant; d, day.

To elucidate the regulatory relationship between Tcirg1 and osteoclast differentiation, we developed an osteoclast differentiation model by stimulating BMMs with M-CSF and RANKL. The cells were stained for Trap and F-actin content (the latter by TRITC-labeled phalloidin staining) to validate the model. The results indicate that BMMs began differentiating into multinucleated Trap-positive osteoclasts by day 3. Differentiation progressed as the cells fused into larger osteoclasts by day 5, and further development was observed by day 7 (Figures 4A,B). RT-qPCR and WB analyses demonstrated that Tcirg1 transcriptional and translational expression increased in a time-dependent manner during osteoclast differentiation (Supplementary Figure S6A; Figures 4C,D). Considering the critical role of lysosomal activity and transport in osteoclasts, using RT-qPCR and WB, we observed that the transcriptional and translational expression levels of Lamp2 (a lysosomal marker) and Rab7 (a regulator of lysosomal transport) gradually increased in a time-dependent manner during osteoclast differentiation (Supplementary Figure S6B,C; Figures 4C,E,F). We further analyzed the co-distribution of Tcirg1 and Lamp2 during various stages of osteoclast differentiation (days 1–7). Confocal microscopy images (Figure 4G) and fluorescence intensity distribution curves (collected along the white lines extending from the center to the periphery of the cells in Figure 4G) suggest a similar intracellular distribution of Tcirg1 and Lamp2 within osteoclasts. In the early stages of differentiation (days 1 and 3), fluorescence intensity for both Tcirg1 and Lamp2 was low (Supplementary Figures S7A,B), in accordance with their lower expression levels. In contrast, on days 5 and 7, there was an increase in fluorescence intensity for both Tcirg1 and Lamp2, characterized by higher fluorescence intensity at the cell periphery (Supplementary Figures S7C,D). These findings suggest that during osteoclast differentiation, Tcirg1 shows a distribution pattern associated with lysosomes and gradually accumulates in peripheral cellular regions.

### 3.5 Knockdown of Tcirg1 inhibits osteoclast fusion and bone resorption function but does not affect actin ring formation

To further explore the regulatory role of *Tcirg1* in osteoclasts, three siRNAs targeting *Tcirg1* were designed. To confirm efficient gene delivery, BMMs were transfected with Cy3-labeled non-targeting siRNA (10–100 nM si-Cy3). After 24 h, more than 80% of the cells interfered with 50 nM si-Cy3 displayed strong cytoplasmic fluorescence, indicating successful transfection (Supplementary Figure S8A,B). Subsequent RT-qPCR and WB revealed that among the three siRNAs, si-Tcirg1-2 achieved the most significant reduction in *Tcirg1* expression, decreasing mRNA levels by approximately 75% and protein levels by approximately 70% (Supplementary Figures S8C–E). This sequence was therefore employed for all subsequent experiments and hereinafter it is indicated as si-Tcirg1.

**FIGURE 5:**
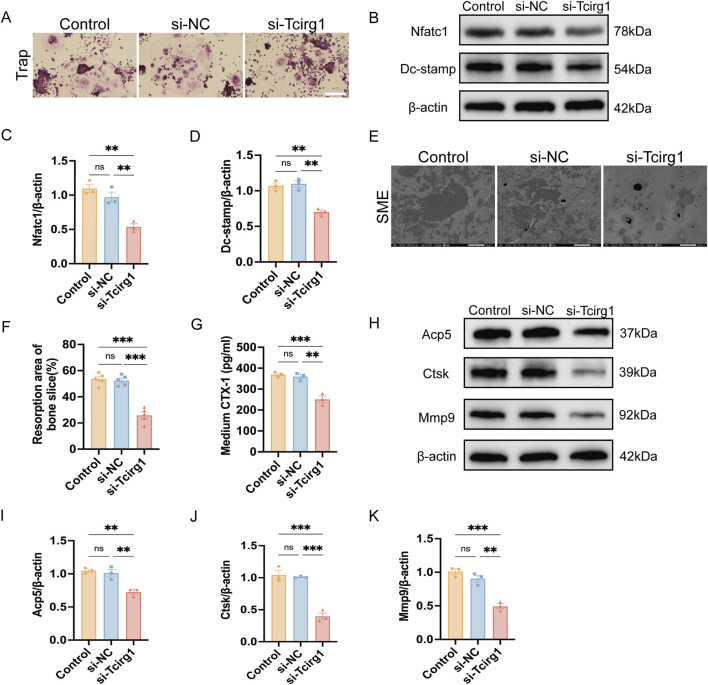
Knockdown of *Tcirg1* inhibits osteoclast fusion and bone resorption. BMMs were untreated (Control) or treated with si-NC/si-Tcirg1 and induced to differentiate using RANKL and M-CSF for 5–7 days. **(A)** Representative Trap staining images; scale bar: 100 μm. **(B)** WB analysis of Nfatc1and Dc-stamp protein expression, markers of osteoclast differentiation and fusion, in different treatment groups. Quantitative analyses of **(C)** Nfatc1 protein expression, **(D)** Dc-stamp protein expression. **(E)** BMMs were untreated (Control) or treated with si-NC/si-Tcirg1, seeded onto homemade bovine bone slices placed in Corning 96-well plates, cultured with M-CSF and RANKL for 7 days, fixed, and processed for SEM; scale bar: 50 μm. **(F)** Quantitative analysis of bone resorption pit areas (calculated as % resorbed mineral surface area, as specified in Methods). **(G)** ELISA detection of CTX-1 protein levels in the culture medium of each group. **(H)** Detection of protein expression of bone resorption markers Acp5, Ctsk, and Mmp9 in different treatment groups using WB. Quantitative analyses of **(I)** Acp5 protein expression, **(J)** Ctsk protein expression, and **(K)** Mmp9 protein expression. Data are expressed as the mean ± SEM (n = 3 per group). Statistical significance was determined using one-way ANOVA followed by Tukey’s *post hoc* test. *P < 0.05, **P < 0.01, ***P < 0.001; ns, not significant.

BMMs were induced to differentiate into osteoclasts using M-CSF and RANKL, and divided into three experimental groups for subsequent analyses: the control group (Control), consisting of untreated cells undergoing differentiation without any siRNA transfection; the negative control siRNA group (si-NC), comprising cells transfected with non-targeting control siRNA; and the Tcirg1 siRNA group (si-Tcirg1). Trap staining revealed that the *Tcirg1* knockdown significantly inhibited the formation of big osteoclasts (Figure 5A). The expression levels of the genes associated with osteoclast differentiation and fusion were examined. *Tcirg1* knockdown significantly downregulated the transcriptional and translational levels of Nfatc1 and Dc-stamp. Specifically, Nfatc1 mRNA levels were reduced by 45% (Supplementary Figure S9A) and its protein levels by 50% (Figures 5B,C), while Dc-stamp mRNA levels decreased by 50% (Supplementary Figure S9B) and its protein levels by 35% (Figures 5B,D) compared to the control group. To evaluate the role of Tcirg1 in osteoclast bone resorption, BMMs were seeded onto homemade bovine bone slices placed in Corning 96-well plates. Following induction of osteoclast differentiation, the bone resorption pit area on the bone slices was assessed using a scanning electron microscope. The results revealed that Tcirg1 knockdown led to a significant reduction in bone resorption pit area, with a 27% decrease compared to the control group (Figures 5E,F). Accordingly, the concentration of CTX-1, a bone resorption marker, in the culture medium was significantly decreased by 31% after *Tcirg1* knockdown (Figure 5G). Consistently, the transcriptional and translational levels of Acp5, Ctsk and Mmp9 (key markers of osteoclastic bone resorption) were significantly reduced following *Tcirg1* knockdown. Specifically, Acp5 mRNA levels decreased by 45% (Supplementary Figure S9C) and its protein levels by 30% (Figures 5H,I); Ctsk mRNA levels dropped by 70% (Supplementary Figure S9D) with a 63% reduction in protein levels (Figures 5H,J); Mmp9 mRNA levels were reduced by 60% (Supplementary Figure S9E) while its protein levels decreased by 45% (Figures 5H,K).

F-actin staining showed that *Tcirg1* knockdown did not affect F-actin ring formation in the osteoclasts (Supplementary Figure S10A). We examined the effect of *Tcirg1* knockdown on the expression levels of αvβ3 integrins (which are involved in actin ring formation). The results of RT-qPCR and WB showed that *Tcirg1* knockdown did not affect the transcription and translation levels of αvβ3 integrin (Supplementary Figures S10B–F). These findings suggested that *Tcirg1* knockdown did not impair osteoclast bone resorption by disrupting actin ring formation.

### 3.6 Knockdown of Tcirg1 impairs lysosomal acidification and peripheral accumulation of lysosomes in osteoclasts

**FIGURE 6:**
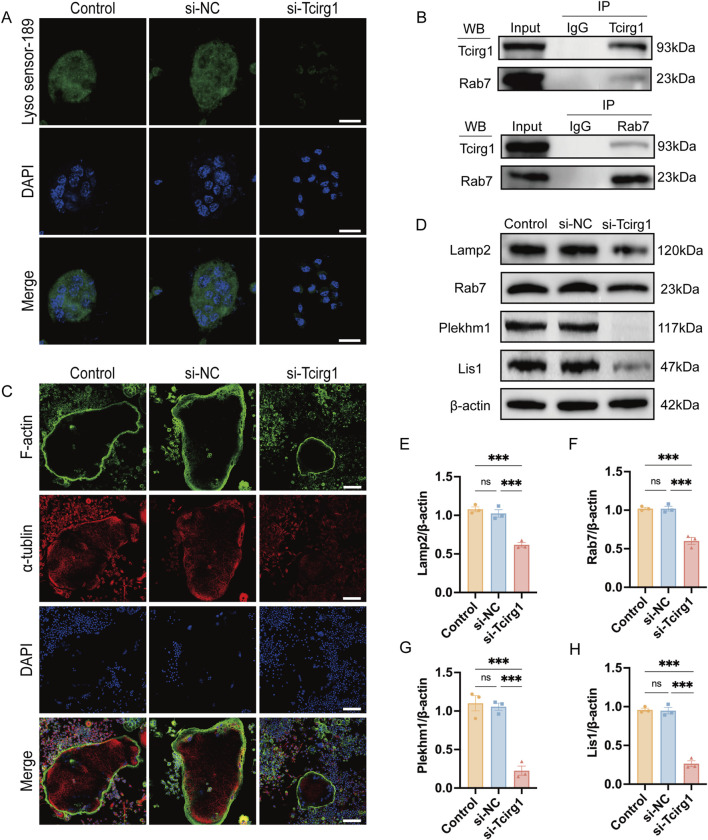
Knockdown of *Tcirg1* impairs lysosomal acidification and microtubule related lysosome distribution in osteoclasts. **(A)** Induction of osteoclastic differentiation of BMMs after their treatment with si- Tcirg1or si-NC, followed by fluorescent staining with LysoSensor™ Green DND-189; scale bar, 20 μm. **(B)** Interaction of Tcirg1 with Rab7 determined using Co-IP with total protein extracts from BMMs. **(C)** Representative images of α-tubulin and F-actin immunofluorescence staining for each treatment group; scale bar, 100 μm. **(D)** WB detection of the protein expression of lysosomal markers Lamp2, Rab7, Plekhm1, and Lis1 in different treatment groups. Quantitative analyses of **(E)** Lamp2, **(F)** Rab7, **(G)** Plekhm1, and **(H)** Lis1 protein expression in different treatment groups. Data are expressed as the mean ± SEM (n = 3 per group). Statistical significance was determined using one-way ANOVA followed by Tukey’s *post hoc* test. ***P < 0.001, and ns. not significant.

To evaluate the impact of *Tcirg1* knockdown on lysosomes, LysoSensor Green DND-189 fluorescent dye was used to measure acidification activity in osteoclasts. A significant reduction in the green fluorescence intensity was observed in osteoclasts following *Tcirg1* knockdown (Figure 6A).

Immunofluorescence analysis was performed to examine the localization of Tcirg1 and Lamp2 in osteoclasts. The results indicated that following *Tcirg1* knockdown, the fluorescence signals of Tcirg1 and Lamp2 were weakened (Supplementary Figure S11A). Then we assed Tcirg1 interaction with Rab7 using a Co-IP assay, which confirmed the binding of these two proteins (Figure 6B). The immunofluorescence analysis revealed Tcirg1 co-distribution with Rab7 at the osteoclast periphery in the control group and in the si-NC groups. However, Rab7 fluorescence signals were notably diminished in *Tcirg1*-knockdown cells, and the remaining signals showed a loss of peripheral accumulation (Supplementary Figure S11B). Microtubules guide lysosomes to the cell periphery to support osteoclast-mediated bone matrix degradation. Microtubule staining revealed a faint α-tubulin staining at the cell periphery in *Tcirg1*-knockdown osteoclasts (Figure 6C), indicating disrupted microtubule organization. We further investigated this by examining the protein levels of Lamp2, Rab7, Plekhm1, and Lis1. WB analysis revealed that following *Tcirg1* knockdown, Lamp2 protein levels decreased by 40%, Rab7 by 40%, Plekhm1 by 80%, and Lis1 by 70% (Figures 6D–H).

## 4 Discussion

In OA, factors such as altered mechanical loading, obesity, and aging impair subchondral bone remodeling by excessively activating osteoclasts, increasing bone resorption, and altering the microstructure (Hu et al., 2021; Zhu et al., 2019). These changes impair the force transmission and accelerate cartilage degeneration. However, the precise mechanism responsible for abnormal osteoclast activation in OA remains unknown. While drugs such as bisphosphonates and denosumab have the potential to reduce osteoclast activity and alleviate OA symptoms, their clinical application is restricted owing to their severe side effects (Laslett et al., 2014; Neogi et al., 2018; Wittoek et al., 2024). These limitations highlight the urgent need to develop novel osteoclast-targeted therapies to effectively slow OA progression. In this study, proteomic analysis of subchondral bone from OA patients revealed significant upregulation of TCIRG1 in WBR—sites characterized by severe cartilage erosion and subchondral bone remodeling—compared to NWBR. This finding was validated by WB and immunohistochemistry. TCIRG1 is well-documented in bone disorders: mutations in *TCIRG1*, account for over 50% of infantile malignant autosomal recessive osteopetrosis cases (Sobacchi et al., 2013), and its R740S mutation in mice disrupts proton pump function, inducing osteopetrosis and highlighting its key role in bone homeostasis (Ochotny et al., 2011). It also shows therapeutic potential in other bone conditions—local injection of a3-specific adeno-associated virus -mediated shRNA protected mice from bone loss and periodontal inflammation induced by *Porphyromonas gingivalis* infection (Jiang et al., 2013). Given the critical role of TCIRG1 in osteoclast function and its involvement in bone-related diseases, we evaluated the impact of Tcirg1 deficiency on OA progression in the DMM-induced mouse model and explored potential mechanisms by *in-vitro* approaches. The DMM model induces OA through destabilization of the meniscus, with trauma as the initiating factor. However, compared to other acute trauma models (e.g., anterior cruciate ligament transection, ACLT), DMM induces a more gradual pathological process: subchondral bone remodeling and cartilage degeneration progress over time, better mimicking the “chronic mechanical imbalance-driven progression” characteristic of human primary OA. In contrast, models like ACLT exhibit more pronounced acute trauma responses, resembling post-acute traumatic OA. This gradual progression makes DMM particularly suitable for our study focusing on long-term OA pathogenesis involving subchondral bone changes (Croen et al., 2023; Glasson et al., 2007). Our findings demonstrated that DMM-induced OA progression was accompanied by a marked rise in osteoclast activity and a corresponding decrease in subchondral bone volume at 4 weeks, mirroring the temporal bone-loss pattern reported by Ding and colleagues (Ding et al., 2022) in the same model. Importantly, *Tcirg1* deficiency in heterozygous knockout mice (Tcirg1^+/−^) reduced osteoclast numbers, preserved subchondral bone mass, and delayed OA progression, without histopathological changes in major organs. These findings indicate that Tcirg1 is a critical regulator of osteoclast formation and function in OA and support its potential as a therapeutic target.

Our study demonstrated that Tcirg1 expression is dynamically upregulated during osteoclast differentiation of BMMs, with prominent localization at the cell periphery—consistent with its potential role in ruffled border formation. Functional validation via siRNA-mediated Tcirg1 knockdown revealed a marked reduction in multinucleated osteoclast formation, accompanied by downregulated expression of Nfatc1 and Dc-stamp, key regulators of osteoclast differentiation and fusion. Osteoclast-mediated bone resorption is a highly coordinated process involving bone matrix adhesion, integrin-dependent cytoskeletal rearrangement, and polarization to form a sealing zone and ruffled border—structures essential for focal resorption (Veis and O’Brien, 2023). The ruffled border, in particular, relies on polarized trafficking of “secretory lysosomes” to the bone-facing plasma membrane, where they deliver V-ATPase and release protons to establish the acidic microenvironment required for inorganic bone dissolution, while secreting hydrolytic enzymes (e.g., Ctsk, Mmp9) to degrade organic matrix components. Previous studies have shown that various V-ATPase subunits have distinct functions: the d2 subunit is highly expressed in mature osteoclasts, essential for maintaining extracellular acidification and participating in pro-osteoclast fusion as a marker of maturation (Lee et al., 2006), while the c1 subunit regulates cytoskeletal F-actin assembly and works closely with the a3 subunit to maintain an acidic environment (Feng et al., 2009). Our data show that *Tcirg1* knockdown severely impaired bone resorption, as evidenced by reduced resorption pit area and downregulated expression of Acp5, Mmp9, and Ctsk. Notably, despite the inhibition of resorptive function, actin ring formation remained intact—consistent with unaltered αvβ3 integrin expression—whereas extracellular acidification capacity was nearly abolished, a phenotype aligning with observations in patients with ATP6i-dependent autosomal recessive osteopetrosis (Taranta et al., 2003). This reinforces that Tcirg1 role in bone resorption is not mediated through cytoskeletal reorganization or adhesion, but rather through processes critical for ruffled border function. Collectively, our findings indicate that Tcirg1 regulates osteoclast resorption through two interconnected mechanisms: first, by maintaining the acidification of secretory lysosomes and resorption lacunae; second, by facilitating lysosomal trafficking to the cell periphery, a step essential for fusion with the ruffled border and subsequent release of hydrolytic enzymes. Disruption of either process ultimately impairs osteoclast-mediated bone matrix degradation.

To clarify how Tcirg1 mediates lysosomal accumulation at the ruffled border, we focused on its interactions with Rab7 and downstream regulatory factors. As key regulators of membrane trafficking, Rab GTPases—particularly Rab7—are central to lysosomal transport in osteoclasts (Itzstein et al., 2011). Localized to late endosomes, lysosomes, and ruffled borders, Rab7 governs endosomal-lysosomal fusion and drives secretory lysosome accumulation at resorptive sites (Guerra and Bucci, 2016). Our Co-IP assays confirmed a direct interaction between Tcirg1 and Rab7 in osteoclasts, consistent with prior observations of a3-Rab7 binding in HEK293T cells (Matsumoto et al., 2018). Functionally, Tcirg1 knockdown disrupted Rab7 peripheral localization, impairing lysosomal accumulation at the cell periphery. Rab7 role in lysosomal transport is further modulated by its interaction with Plekhm1 (Ng et al., 2019), whose mutations are linked to osteosclerosis (Sobacchi et al., 2013); Plekhm1 deficiency disrupts lysosomal anchoring to microtubules, impairing bone resorption (Fujiwara et al., 2016). Additionally, Plekhm1 binds Lis1 via its “RPIP8/UNC-14/NESCA” (RUN) and “pleckstrin homology 1” (PH1) domains, linking lysosomes to microtubules, while Lis1 stabilizes microtubule dynamics via dynein-dynactin interactions to ensure directional lysosomal transport (Ye et al., 2011; Singh et al., 2024). In our study, Tcirg1 knockdown reduced Rab7, Plekhm1, and Lis1 expression, concurrent with disorganized microtubules at the cell periphery. Collectively, these data support a model wherein Tcirg1, through its interaction with Rab7, modulates Plekhm1 and Lis1 expression to stabilize the microtubule network, thereby facilitating lysosomal peripheral enrichment and subsequent ruffled border formation—critical steps for sustaining osteoclast resorptive activity.

Notably, Tcirg1 role in OA may extend beyond direct regulation of osteoclast resorption. Podosomes—unique cytoskeletal structures in osteoclasts consisting of an F-actin core and surrounding adhesion proteins that provide mechanical stability and adhesive capacity (Dufrançais et al., 2021; Chen et al., 2023)—rely on Tcirg1 for their formation and function, with their structural integrity essential for Tcirg1-mediated lysosomal peripheral accumulation and acidification abnormalities in OA could disrupt bone resorption balance, exacerbating cartilage degeneration, warranting further study on Tcirg1-podosome interactions as a potential therapeutic target. Additionally, Tcirg1 may modulate osteoclast activity via autophagy: Rab7, a key component in the Tcirg1 pathway, is critical for lysosomal autophagy linked to osteoclast function, as observed in zebrafish where Tcirg1 deletion impaired autophagy, as evidenced by enhanced LC3-I to LC3-II conversion and accumulation of autophagosomes (Chen et al., 2024). Meanwhile, Plekhm1 bridges lysosomes to autophagy pathways and synergizes with Rab7 to facilitate efficient lysosomal transport and bone resorption (McEwan et al., 2015). Lysosomal dysfunction from Tcirg1 impairment could also activate the NLRP3 inflammasome, promoting pro-inflammatory cytokine release (Xia et al., 2019) and exacerbating OA-related inflammation. Metabolically, Tcirg1 mutations disrupt gastric parietal cell V-ATPase function, impairing calcium absorption, inducing hypocalcemia, and stimulating PTH secretion—altering insulin sensitivity, glucose metabolism (Sobacchi et al., 2013), and 1,25-dihydroxyvitamin D3 levels, potentially disrupting chondrocyte energy metabolism and accelerating OA. These interconnected pathways merit exploration to fully elucidate Tcirg1’s role in OA’s multifactorial pathogenesis. A limitation of our study is the use of plastic substrates for *in vitro* osteoclast cultures, restricting direct visualization of ruffled border formation and lysosomal dynamics; future studies using bone slices or 3D culture systems will better clarify lysosomal peripheral accumulation and its dynamic association with ruffled border formation in Tcirg1-deficient osteoclasts, while osteoclast-specific conditional knockout mouse models could further clarify cell-type-specific effects.

**FIGURE 7:**
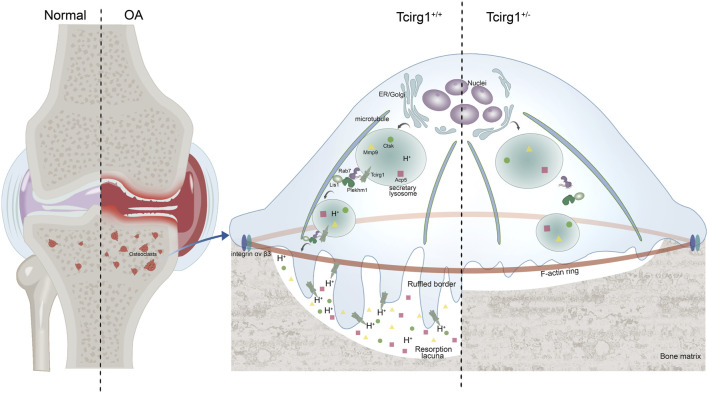
Schematic diagram of *Tcirg1* knockout on OA. Tcirg1 maintains lysosomal acidification, and influences the expression of Rab7, Plekhm1, and Lis1, enabling lysosomes to localize and accumulate at the cell periphery in a microtubule - dependent manner. This promotes the formation of ruffled borders and further ensures the maintenance of osteoclast bone resorption activity. Knocking down Tcirg1 significantly reduces osteoclast activity, which in turn limits abnormal subchondral bone remodeling and cartilage damage in OA.

In conclusion, our study demonstrates that Tcirg1 critically regulates osteoclast-mediated bone resorption by coordinating lysosomal acidification and modulating Rab7, Plekhm1, and Lis1 expression. This regulation drives microtubule-dependent lysosomal localization and accumulation at the cell periphery—a prerequisite for ruffled border formation and efficient bone matrix degradation. In a murine OA model, Tcirg1 deficiency impairs osteoclast activity, alleviates subchondral bone remodeling abnormalities, reduces cartilage damage, and delays disease progression (Figure 7). These findings identify Tcirg1 as a key regulator of osteoclast function in OA pathogenesis and a promising therapeutic target.

## Data Availability

The original contributions presented in the study are included in the article/Supplementary Material, further inquiries can be directed to the corresponding authors.

## Abbreviations

Tcirg1, T-cell immune regulator 1; OA, Osteoarthritis; WBR, weight-bearing region; NWBR, non-weight-bearing region; H&E, hematoxylin and eosin; Mmp9, Matrix metalloproteinase 9; Ctsk, Cathepsin K; Lamp2, Lysosome-associated membrane glycoprotein 2; Rab7, Ras-related protein Rab-7; Plekhm1, Pleckstrin homology domain-containing family M member 1; Lis1, Lissencephaly-1 homolog; RANKL, Receptor Activator of nuclear factor-κB Ligand; M-CSF, Macrophage-colony stimulating factor; BMM, Bone marrow macrophage; DMM, destabilization of the medial meniscus; OARSI, Osteoarthritis Research Society International; Trap, tartrate-resistant acid phosphatase; Acp5, Tartrate-resistant acid phosphatase type 5; Nfatc1, Nuclear factor of activated T-cell cytoplasmic 1; Dc-stamp, Dendritic cell specific transmembrane protein; Micro-CT, Micro-computed tomography; BV/TV, bone volume/total volume; Tb.Th, trabecular thickness; Tb.Sp, trabecular separation; WB, western blotting.
