# Supplementary material for: Tcirg1 deficiency delays osteoarthritis progression by impairing lysosome acidification and peripheral accumulation in osteoclasts
Source: Front Cell Dev Biol. 2025 Sep 9;13:1621648. doi: 10.3389/fcell.2025.1621648 (PMC12454451; doi:10.3389/fcell.2025.1621648)
Supplement: Supplementary file 1 [file DataSheet1.docx]

Supplementary Material

# Supplementary Figures and Tables

## Supplementary Figures

**
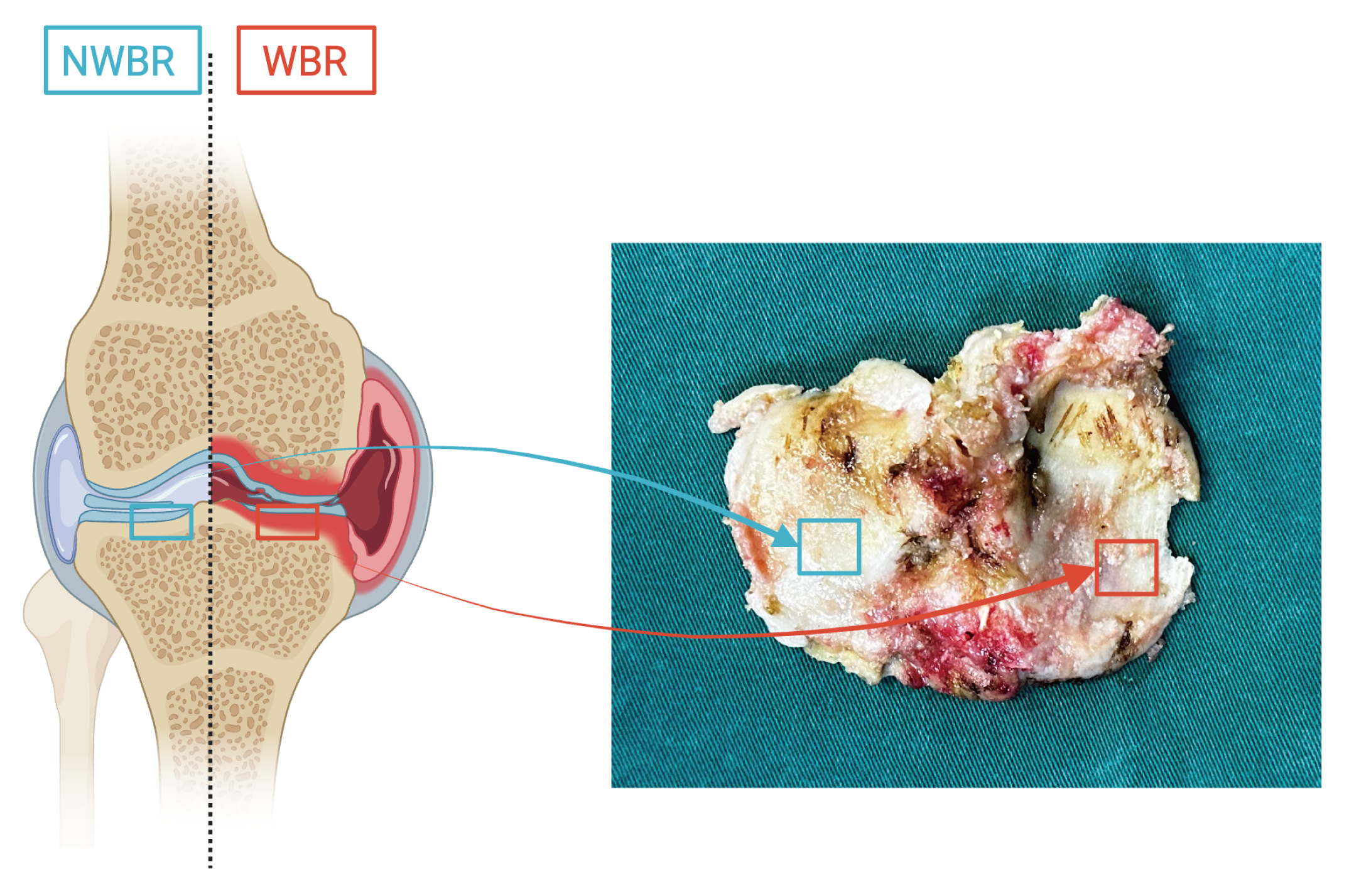
**

**Supplementary Figure 1.** Schematic diagram showing the anatomical regions of the tibial plateau in the knee joint of OA patients. The left side presents a cross - sectional illustration of the knee joint. The dashed line demarcates the tibial plateau into two functional regions: the ‘non-weightbearing region’ (NWBR, labeled in cyan), which corresponds to the lateral tibial plateau, and the ‘weight-bearing region’(WBR, labeled in red), representing the medial tibial plateau that undertakes weight - bearing tasks. The right side displays the actual resected tibial plateau tissue specimen from OA patients.

**
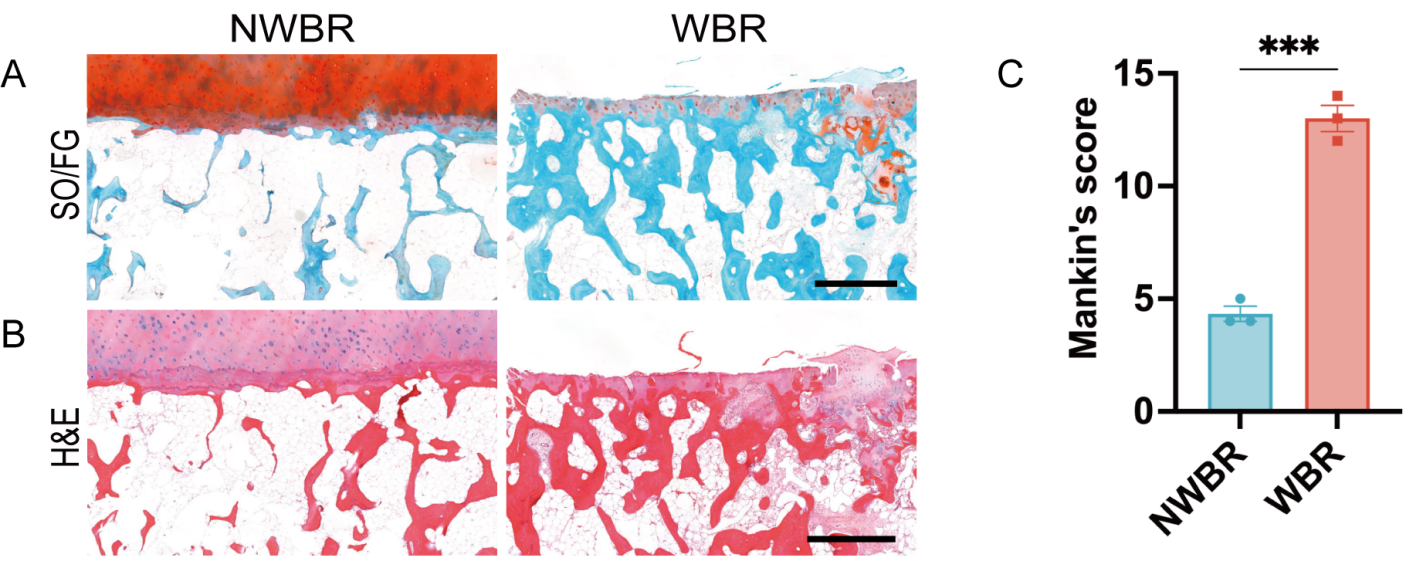
**

**Supplementary Figure 2.** Assessment of OA pathology in WBR/NWBR subchondral bone and cartilage regions. (A) SO/FG, and (B) H&E staining of subchondral bone and cartilage in the tibial plateau. Scale bars, 500 μm. (C) Mankin’s score evaluation, data are expressed as mean ±SEM (n = 3 per group). Statistical significance was determined using an unpaired two-tailed Student’s *t*-test. ***P < 0.001. SO/FG, safranin-O/fast green; H&E, hematoxylin and eosin.


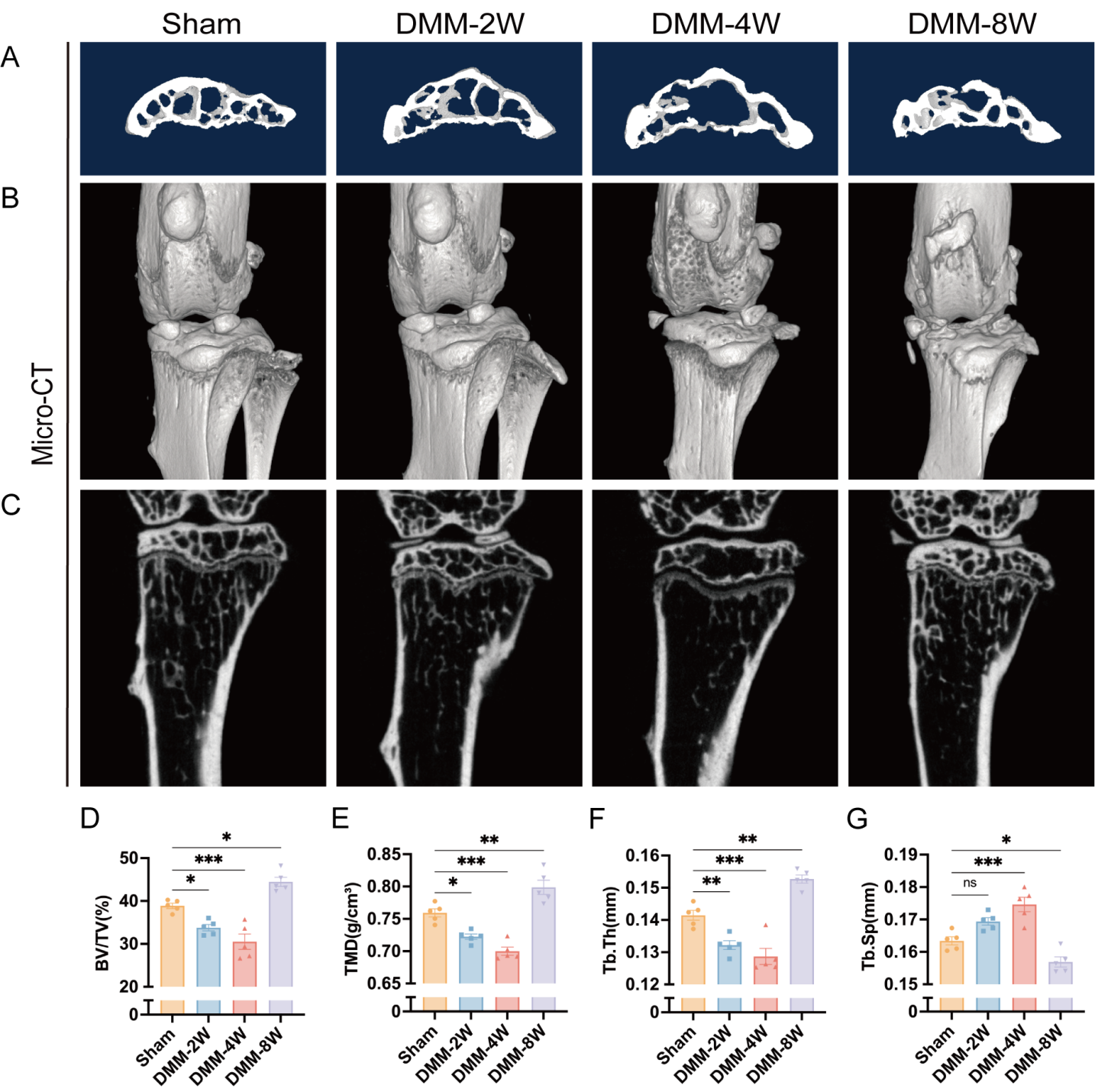


**Supplementary Figure 3.** Dynamic subchondral bone remodeling in DMM-induced OA progression of WT mice. (A-C) Micro-CT analysis of mouse knee joints at 2-, 4-, and 8-weeks after DMM surgery. (A) 2D reconstructed sections; (B) 3D surface renderings; (C) 2D cross-sectional views. Magnification: 10×. Statistical analysis of bone morphometric parameters: (D) BV/TV, (E) TMD, (F) Tb.Th, and (G) Tb.Sp. Data are expressed as the mean ±SEM (n = 5 per group). Statistical significance was determined using one-way ANOVA followed by Tukey’s post-hoc test. *P < 0.05, **P < 0.01, ***P < 0.001; ns, not significant.


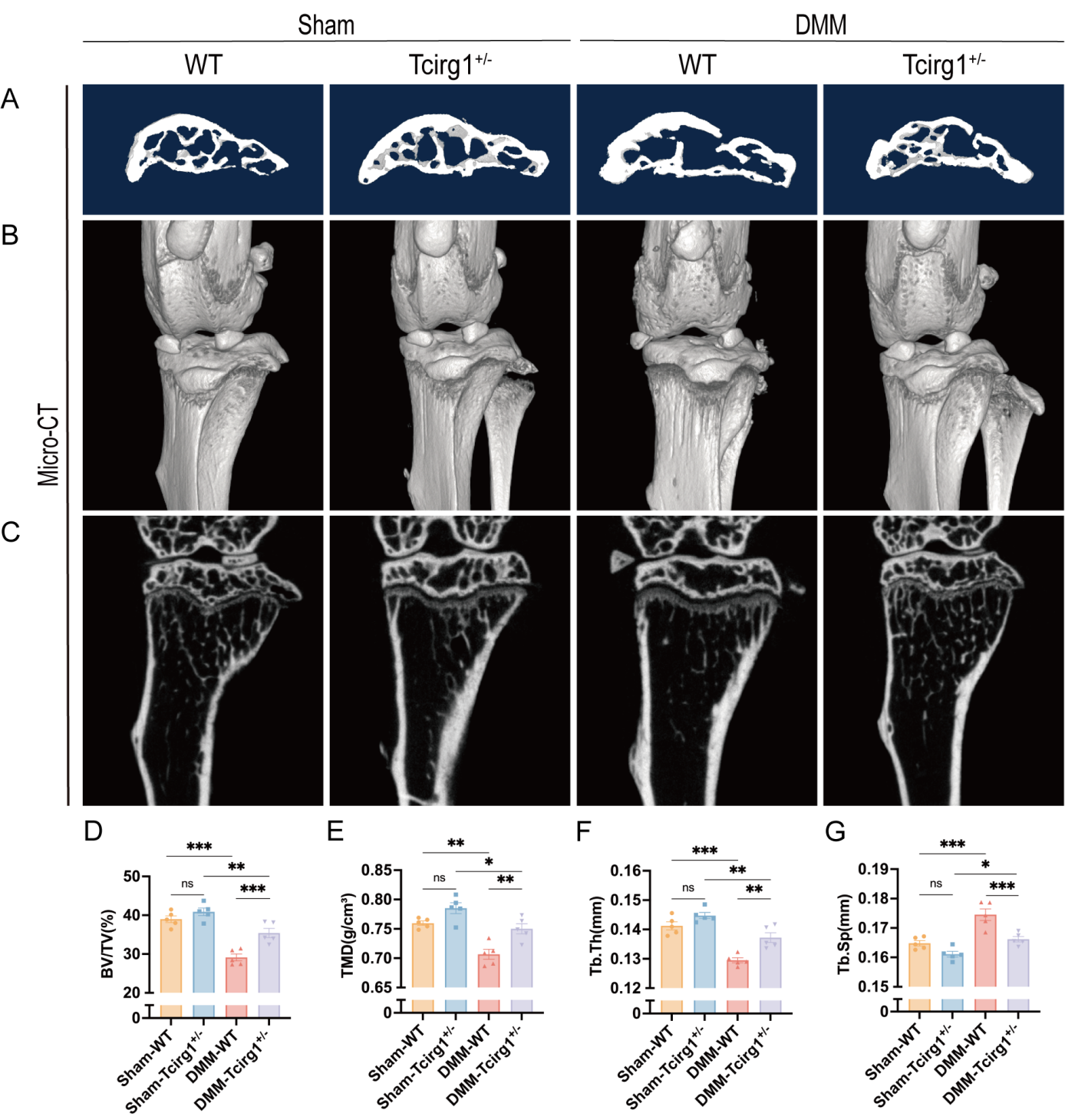


**Supplementary Figure 4.** *Tcirg1* deficiency alleviates OA subchondral bone remodeling in mice. (A-C) Micro-CT analysis of mouse knee joints at 4 weeks after DMM surgery. (A) 2D reconstructed sections; (B) 3D surface renderings; (C) 2D cross-sectional views. Magnification: 10×. Statistical analysis of bone morphometric parameters: (D) BV/TV, (E) TMD, (F) Tb.Th, and (G) Tb.Sp. Data are expressed as the mean ± SEM (n = 5 per group). Statistical significance was determined using one-way ANOVA followed by Tukey’s post-hoc test. *P < 0.05, **P < 0.01, ***P < 0.001; ns, not significant.

**
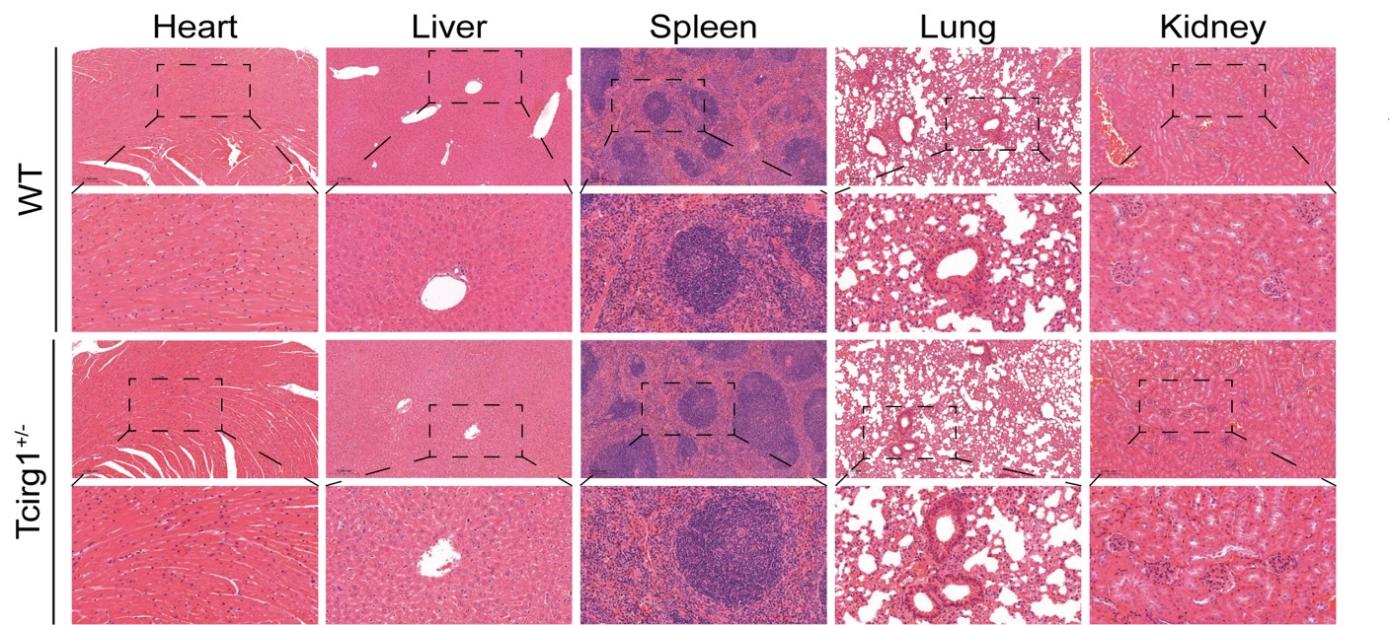
**

**Supplementary Figure 5.** Effects of *Tcirg1* deletion on the heart, liver, spleen, lungs and kidneys of mice. Representative images of H&E staining of heart, liver, spleen, lungs, and kidneys from WT mice and *Tcirg1*-deficient mice. Magnification: 15× first and third row, and 40× boxed images in second and fourth row of images.

**
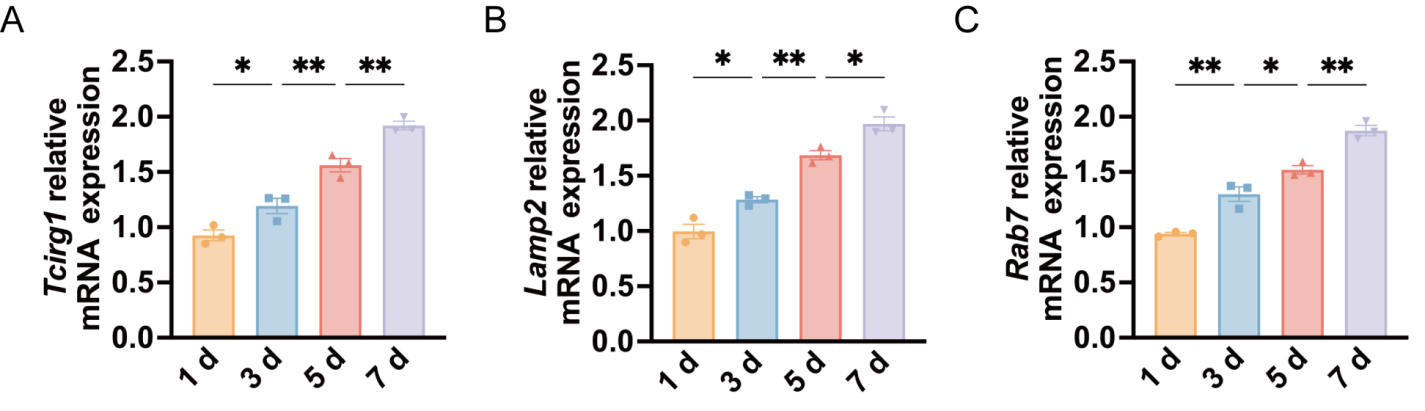
**

**Supplementary Figure 6.** Temporal expression dynamics of Tcirg1, Lamp2, and Rab7 during osteoclast differentiation. Quantitative analysis of (A) Tcirg1 mRNA expression, (B) Lamp2 mRNA expression, and (C) Rab7 mRNA expression. Data are expressed as the mean ±SEM (n = 3 per group). Statistical significance was determined using one-way ANOVA followed by Tukey’s post-hoc test. *P < 0.05, **P < 0.01; d, day.


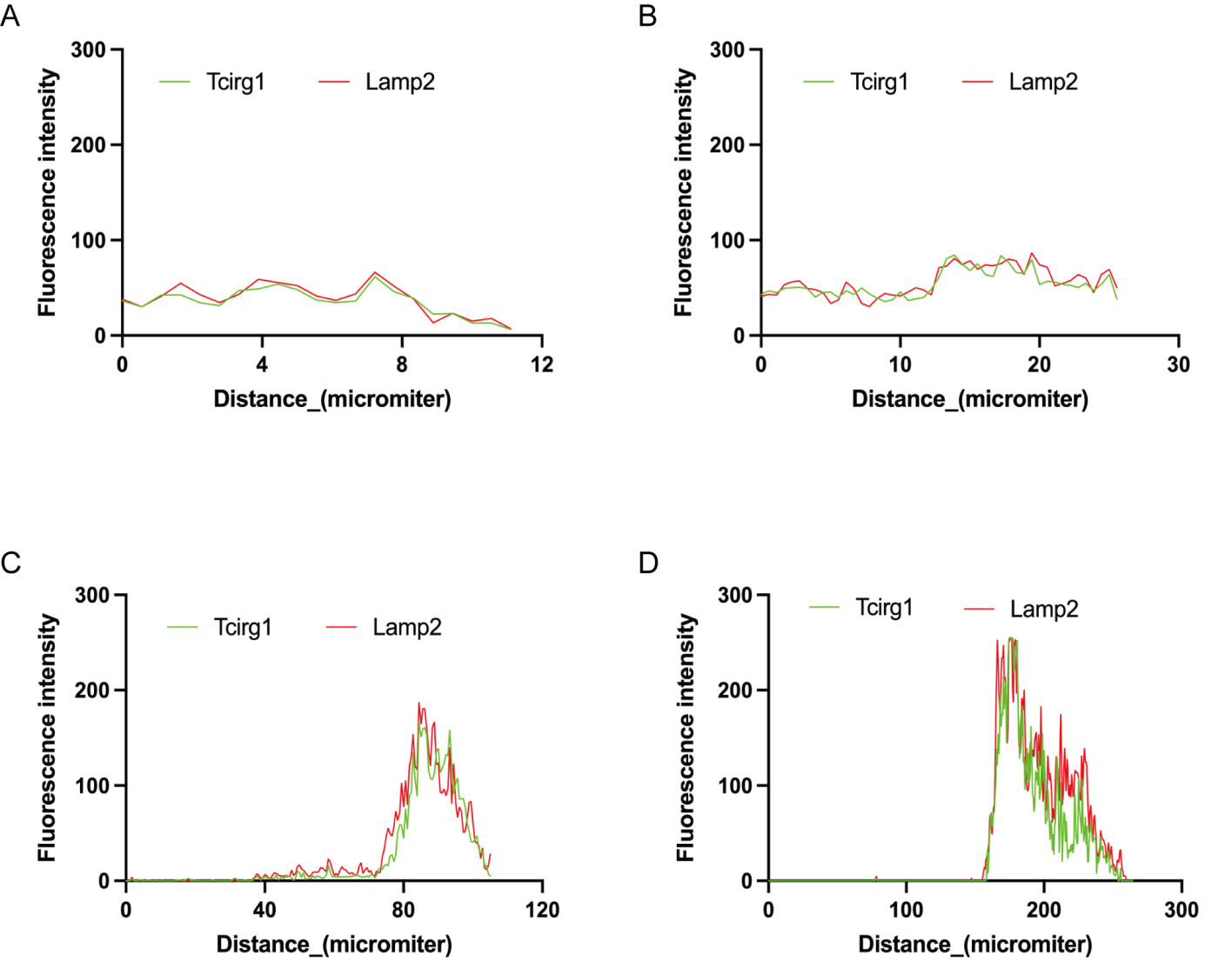


**Supplementary Figure 7.** Fluorescence intensity distribution curves of Tcirg1 and Lamp2. For their co-distribution analysis Figure 4G, Panels A, B, C, and D correspond to 1, 3, 5, and 7 days, respectively. In all the graphs, The X - axis represents the distance from the cell center to the edge along the short axis of osteoclasts, and the Y - axis represents the fluorescence intensity. The green line indicates Tcirg1, while the red line represents Lamp2 fluorescence signals.


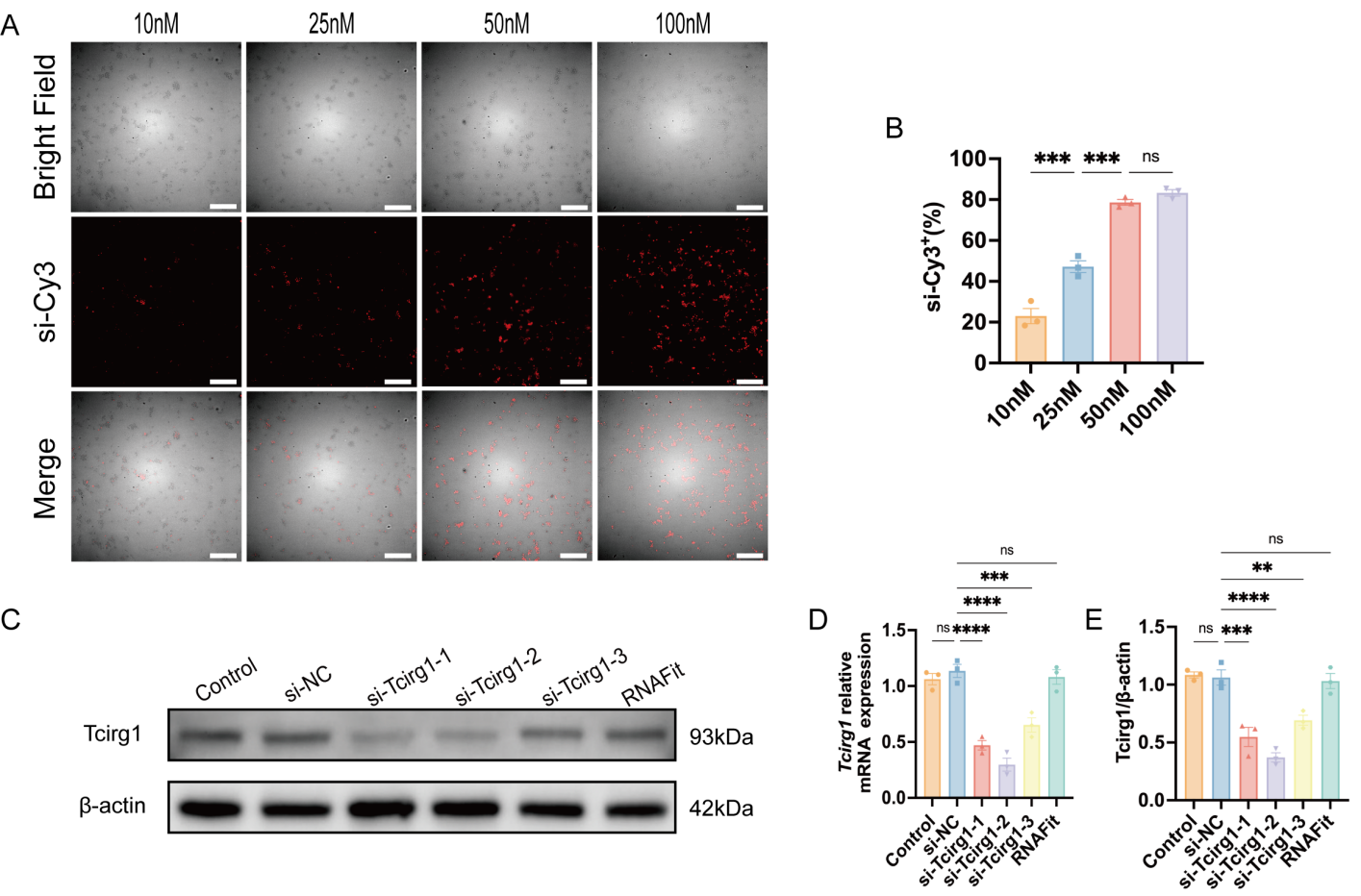


**Supplementary Figure 8.** Quantification of transfection and knockdown efficiency. (A) Representative bright field and fluorescence images after transfection with different concentrations of si-Cy3. Scale bars, 200 μm. (B) Quantification of transfection efficiency as percentage of si-Cy3 positive cells. (C) BMMs were left untreated (Control) or treated with a negative control (si-NC), or three different anti-Tcirg1 siRNAs (si- Tcirg1), or by the transfection reagent alone (RNAFit), thereafter Tcirg1 knockdown efficiency was assessed using WB after 3 days. Quantitative analyses of (D) Tcirg1 mRNA levels and (E) protein levels under different conditions. Data are expressed as the mean ±SEM (n = 3). Statistical significance was determined using one-way ANOVA followed by Tukey’s post-hoc test. **P < 0.01, ***P < 0.001, ****P < 0.0001; ns, not significant.


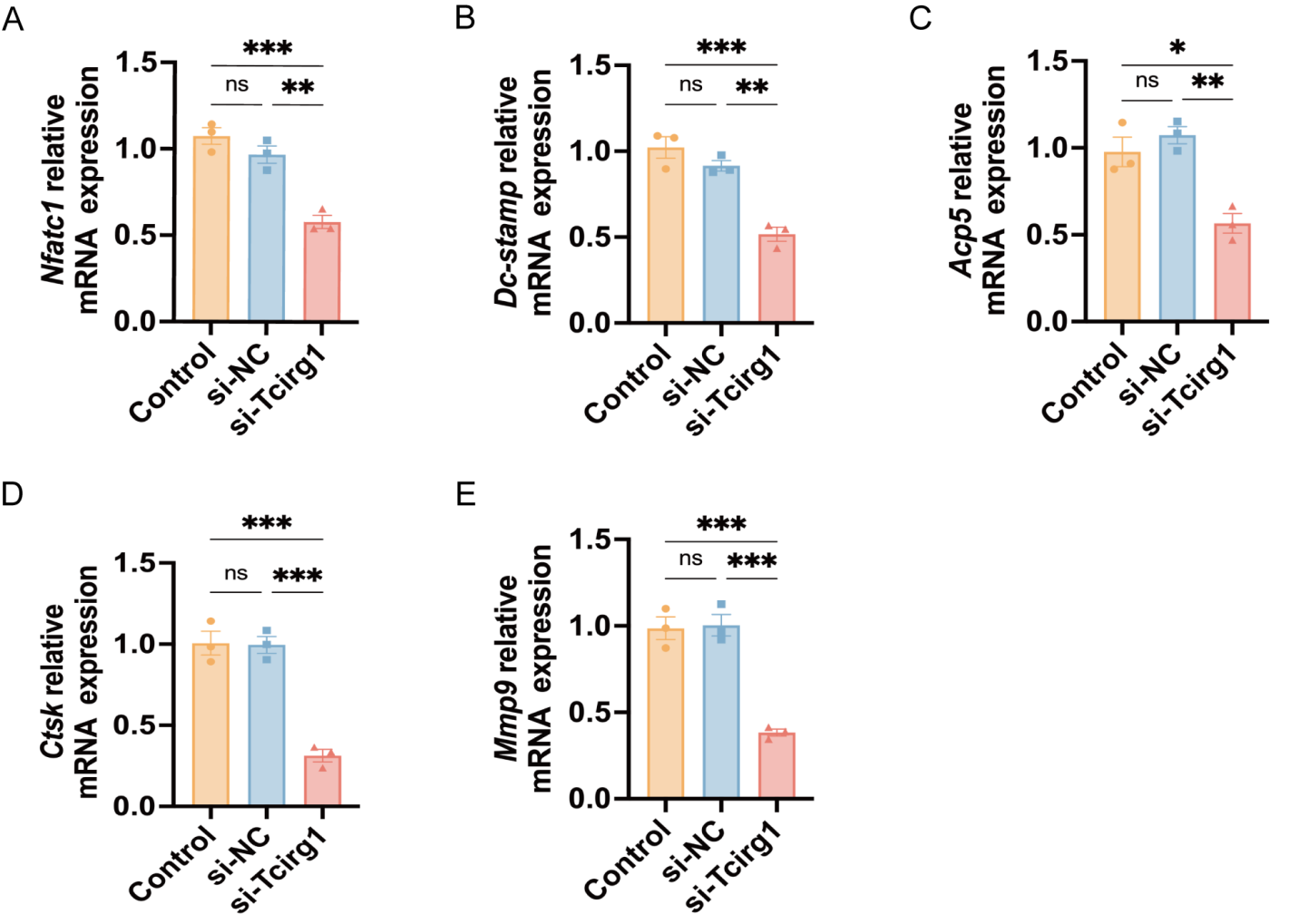


**Supplementary Figure 9.** Effects of Tcirg1 knockdown on markers of osteoclast fusion, bone resorption. Quantitative analysis of (A) Nfatc1 mRNA expression, (B) Dc-stamp mRNA expression, (C) Acp5 mRNA expression, (D) Ctsk mRNA expression, and (E) Mmp9 mRNA expression. Data are expressed as the mean ± SEM (n = 3 per group). Statistical significance was determined using one-way ANOVA followed by Tukey’s post-hoc test. *P < 0.05, **P < 0.01, ***P < 0.001, ns: not significant.


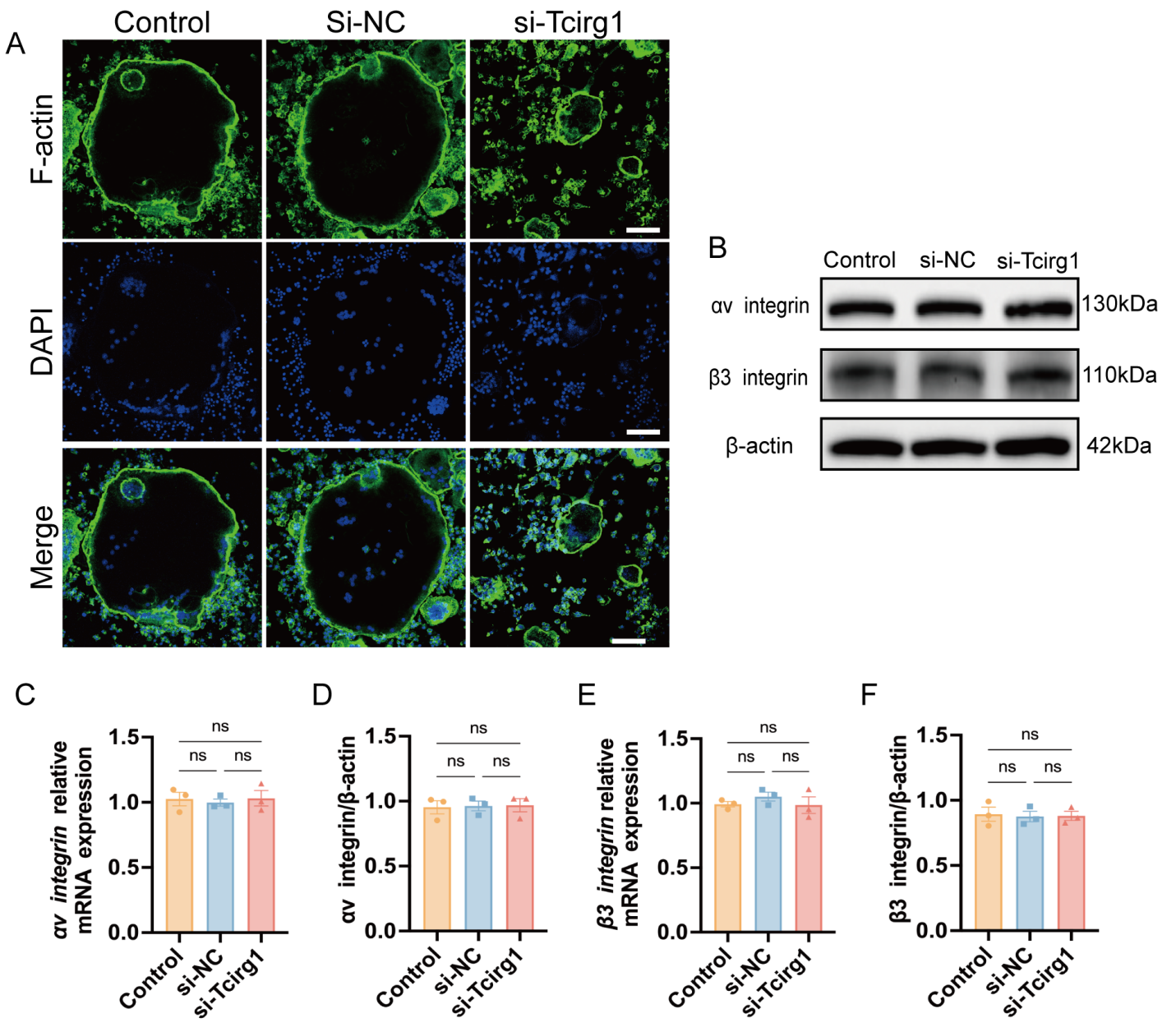


**Supplementary Figure 10.** Knockdown of Tcirg1 does not affect osteoclast actin ring formation. BMMs were untreated (Control) or treated with si-NC/si-Tcirg1 and induced to differentiate using RANKL and M-CSF for 5–7 days. (A) Representative images of F-actin and DAPI staining. Scale bars, 100 μm. (B) WB analysis of αv and β3 integrin protein expression in different treatment groups. Quantitative analyses of (C) αv integrin mRNA and (D) protein expression, and (E) β3 integrin mRNA and (F) protein expression. Data are expressed as the mean ±SEM (n = 3 per group). Statistical significance was determined using one-way ANOVA followed by Tukey’s post-hoc test. ns, not significant.


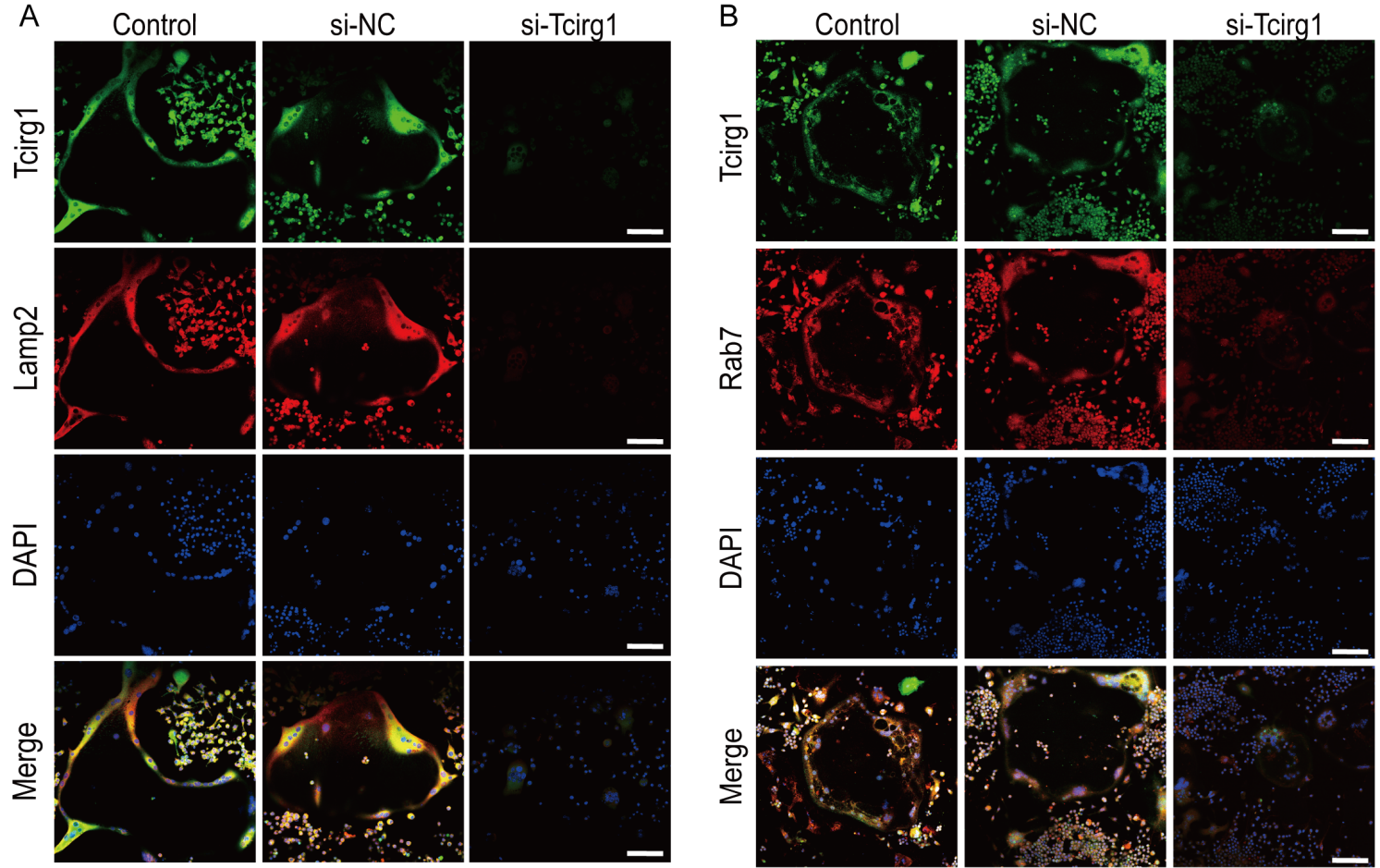


**Supplementary Figure 11.** Altered localization of Tcirg1, Lamp2, and Rab7 in Tcirg1 knocked down osteoclasts. Representative images (A) of Tcirg1 and Lamp2, and (B) of Tcirg1 and Rab7 immunofluorescence staining for each treatment group. Scale bars, 100 μm.

## Supplementary Tables

**Supplementary Table 1.** si-RNA sequences.

| si-RNA name | Primer sequences (5' - 3') |
| --- | --- |
| si-Tcirg1-1 | Forward: GAACUGGAGAAGACGUUUATT |
|  | Reverse: UAAACGUCUUCUCCAGUUCTT |
| si-Tcirg1-2 | Forward: CAUUCAGACCUGAAGGUCATT |
|  | Reverse: UGACCUUCAGGUCUGAAUGTT |
| si-Tcirg1-3 | Forward: CUCAUUCACUUCAUCAAUATT |
|  | Reverse: UAUUGAUGAAGUGAAUGAGTT |
| si-NC | Forward: UUCUCCGAACGUGUCACGUTT |
|  | Reverse: ACGUGACACGUUCGGAGAATT |
| si-Cy3 | Forward: UUCUCCGAACGUGUCACGUTT |
|  | Reverse: ACGUGACACGUUCGGAGAATT |

**Supplementary Table 2.** Real-time PCR primers.

| Gene symbol (Mus) | Primer sequences (5' - 3') |
| --- | --- |
| Tcirg1 | Forward: GGCTACCGTTCCTATCCT |
|  | Reverse: CTTGTCCGTGTCCTCATC |
| Lamp2 | Forward: CTGCCACAACCAACTTCA |
|  | Reverse: GACATTCACTTCCTTCAGATAG |
| Rab7 | Forward: CACAATAGGAGCGGACTT |
|  | Reverse: ACACCAGAACACAGCAAT |
| Nfatc1 | Forward: CCGAGGAAGAACACTACAG |
|  | Reverse: GGATGATTGGCTGAAGGAA |
| Dc-Stamp | Forward: TTGTGGCTGGAAGTATGAG |
|  | Reverse: CAGGACAGGAAGGAAGAAC |
| Acp5 | Forward: AGTATCTTCAGGACGAGAAC |
|  | Reverse: TCCAGAGGCTTCCACATA |
| Mmp9 | Forward: ACTCACACGACATCTTCC |
|  | Reverse: ATGGTCCACCTTGTTCAC |
| Ctsk | Forward: TTGTGACCGTGATAATGTGA |
|  | Reverse: GCAGGCGTTGTTCTTATTC |
| αv integrin | Forward: AAGGCTGGAACTCAACTG |
|  | Reverse: AGACACAACTGGACTTACAT |
| β3 integrin | Forward: CTACAAGAACGAGGATGACT |
|  | Reverse: GCAGGACCACCAGGATAT |
| Gadph | Forward: TCTCCTGCGACTTCAACA |
|  | Reverse: TGTAGCCGTATTCATTGTCA |
